# Lithium Exposure Causes Trophoblast Cuproptosis by Upregulating FOXO1/STEAP4 Axis in Unexplained Miscarriage

**DOI:** 10.1002/advs.202502139

**Published:** 2025-08-25

**Authors:** Shuaishuai Xing, Yanbing Lin, Yi Sun, Xiaoping Yue, Qigang Fan, Jisheng Nie, Zhihong Zhang, Yajing Liu, Juntang Yang, Qingzheng Kang, Yanxin Wang, Haijun Yan, Chan Tian, Ying Chang, Huidong Zhang

**Affiliations:** ^1^ Research Center for Environment and Female Reproductive Health the Eighth Affiliated Hospital Sun Yat‐sen University Shenzhen 518033 China; ^2^ Key Laboratory of Pharmaceutical Quality Control of Hebei Province College of Pharmaceutical Sciences Hebei University Baoding 071002 China; ^3^ MOE Key Laboratory of Coal Environmental Pathogenicity and Prevention Shanxi Medical University Taiyuan 030001 China; ^4^ School of Public Health Shanxi Key Laboratory of Birth Defect and Cell Regeneration Shanxi Medical University Taiyuan 030001 China; ^5^ Reproductive Medicine Center Department of Obstetrics and Gynecology The First Affiliated Hospital of Anhui Medical University Hefei 230022 China; ^6^ Henan Normal University College of Life Science 46 Jianshe Road Xinxiang Henan 453007 China; ^7^ State Key Laboratory of Female Fertility Promotion Center for Reproductive Medicine Department of Obstetrics and Gynecology Peking University Third Hospital Beijing 100191 China; ^8^ Tianjin Central Hospital of Obstetrics and Gynecology Tianjin 300100 China

**Keywords:** cuproptosis, FOXO1, Li exposure, STEAP4, trophoblast cells, unexplained miscarriage

## Abstract

Lithium (Li) batteries have been used worldwide, but few are recycled, thus, the waste Li has been widely spread into the environment and finally accumulated in human body. Cuproptosis is a newly reported Cu‐dependent and programmed cell death form. The unclear pathogenesis of unexplained miscarriage (UM) largely restricts its clinical treatment and global human reproduction. In this study, a UM case‐control study shows that Li levels in serum or villous tissues, the levels of cuproptosis in villous tissue, and UM are positively associated. Li‐exposed mouse models further confirm that Li exposure causes placental cuproptosis to induce miscarriage. Mechanistically, Li exposure up‐regulates FOXO1 expression levels and thus promotes FOXO1‐mediated STEAP4 transcription, up‐regulating STEAP4 levels. STEAP4 up‐regulates intracellular Cu^+^ ion levels and causes cuproptosis, which further induces miscarriage. The cellular mechanisms are consistent with those in UM villous tissues and Li‐exposed mouse placental tissues. Finally, treatment with TTM to suppress cuproptosis or the therapeutic down‐regulation of FOXO1 or STEAP4 could efficiently suppress placental cuproptosis and alleviate mouse miscarriage in the Li‐exposed mouse models. Collectively, this study not only discovers new healthy risks of Li exposure and novel pathogenesis of Li‐induced unexplained miscarriage but also reveals new biological targets for treatment against miscarriage.

## Graphical abstract

Li exposure up‐regulates FOXO1 expression levels and thus promotes FOXO1‐mediated STEAP4 transcription, up‐regulating STEAP4 levels. Subsequently, STEAP4 up‐regulates intracellular Cu^+^ ion levels and causes cuproptosis, which further induces miscarriage. Treatment with TTM to suppress cuproptosis or the therapeutic down‐regulation of FOXO1 or STEAP4 could efficiently suppress placental cuproptosis and alleviate Li‐exposed mouse miscarriage.

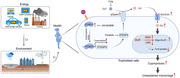

## 1 Introduction

### 1.1 Unexplained Miscarriage (UM)

Approximately 15%–25% pregnant women end with miscarriage, and 1%–5% suffer from recurrent miscarriage in the world,^[^ ^1^ ^]^ which greatly limits global reproduction. Moreover, 41.2% recurrent miscarriage patients have long‐term anxiety, 8.6% have severe depression, and 1.4% even threaten life safety.^[^ ^2^ ^]^ The causes of miscarriage are very complicated, including genetic factors, chromosomal abnormalities, infections, prothrombotic states, abnormal uterine anatomy, endocrine, metabolic, and reproductive immune diseases. However, there are still 50% miscarriages with unknown causes, which are generally termed as unexplained miscarriage.^[^ ^3^, ^4^ ^]^ As the key component of the placenta, trophoblast cells play vital roles in embryo implantation and healthy pregnancy.^[^ ^5^, ^6^ ^]^ Trophoblast cell dysfunctions always induce miscarriage.^[^ ^7^ ^]^ To explore what might cause trophoblast cell dysfunctions and then induce miscarriage is essential for understanding the pathogenesis of unexplained miscarriage.

### 1.2 Lithium (Li) Exposure and Miscarriage

Increasing evidence has demonstrated that environmental toxicants might be important risk factors for unexplained miscarriage.^[^ ^8^, ^9^, ^10^, ^11^, ^12^, ^13^, ^14^, ^15^ ^]^ In recent years, Li exposure has become an emerging important issue in the energy and environment field. Globally, Li products reached 100 000 tons in 2021, 256% more than in 2010^16^, and will be increasing over the next two decades.^[^ ^16^ ^]^ Notably, only 5% Li batteries are recovered at present, and up to 95% waste Li batteries are discarded into the environment.^[^ ^17^ ^]^ In China, Li battery products have experienced explosive growth since 2015, and as predicted, the first wave of Li battery scrap peak will occur in 2026. In addition, Li is also present in global coals (≈12 mg kg) and is released into the atmosphere through combustion.^[^ ^18^ ^]^ It has been reported that ≈5.5 × 10^10^ Li fluxes enter the atmosphere in the world in 2019^15^. As a result, Li contamination is widely present in air, soils, and water. It has been detected that Li content in soils is 17.1–38.5 mg kg^−1^ in Beijing, China,^[^ ^19^ ^]^ 6.4–15 mg kg^−1^ in Europe,^[^ ^20^ ^]^ and 7–200 mg kg^−1^ in Romania.^[^ ^21^ ^]^ In water, Li content is 0.07–40 µg L^−1^ in fresh water, 0–219 µg L^−1^ in drinking water, and 170–190 µg L^−1^ in seawater.^[^ ^22^ ^]^ The level of Li could be increased up to 11.8–13.7 mg L^−1^ in Donbass rivers in Ukraine related to huge volumes of mine wastewater discharges.^[^ ^23^ ^]^ Eventually, the widespread Li is inevitably ingested by the human body through air, food, and drinking water. It has been reported that Li level is 1.9–145 µg L^−1^ in blood and 105–4600 µg L^−1^ in urine^[^ ^24^ ^]^ in the normal population. Moreover, bipolar disorder patients might take 600–1200 mg day^−1^ Li_2_CO_3_ as medicine,^[^ ^25^ ^]^ giving 0.6–1.2 mM Li in their body fluids.^[^ ^26^, ^27^ ^]^ The highly accumulated Li in the body might produce a health risk in humans, which should be urgently investigated.

It has been reported that high levels of Li are toxic to the human muscle, cardiovascular, gastrointestinal, urinary, and nervous systems, and even cause death.^[^ ^28^ ^]^ Short‐term exposure to Li leads to nephrogenic diabetes insipidus, polyuria, polydipsia, dehydration, urinary concentration defects, and thirst; long‐term exposure to Li increases the odds of end‐stage renal disease.^[^ ^29^ ^]^ Recently, emerging evidence has shown that Li exposure exhibits reproductive toxicity. A prospective mother‐child cohort (N = 194) shows that Li levels in maternal blood and urine are inversely associated with fetal measurements.^[^ ^30^ ^]^ Another cohort of women with bipolar I disorder shows that Li intake during pregnancy may increase risk for miscarriage (OR = 2.94, 95% CI: 1.39–6.22).^[^ ^31^ ^]^ A meta‐analysis also showed that Li exposure during the first trimester is associated with miscarriage (N = 1289, k = 3, prevalence = 8.1%; OR = 3.77, 95% CI = 1.15–12.39; NNH = 15, 95% CI = 8–111).^[^ ^32^ ^]^ Moreover, animal model assays also show that Li exposure leads to ovarian steroid hormone synthesis disorder, follicular dysplasia, and premature ovarian failure, which cannot be recovered even after stopping Li exposure.^[^ ^25^, ^26^, ^33^ ^]^ Prenatal Li exposure also causes abnormal embryonic development.^[^ ^27^, ^34^, ^35^ ^]^ Therefore, these preliminary studies have shown that Li exposure could cause reproductive toxicity. However, whether and how Li exposure might induce unexplained miscarriage is still largely unknown and should be fully explored.

### 1.3 Cuproptosis

Cuproptosis is a newly reported Cu‐dependent and programmed cell death, which is different from other known cell death, such as apoptosis, necroptosis, or ferroptosis^[^ ^36^ ^]^. Intracellular Cu⁺ ions interact with fatty acylase, leading to proteotoxic stress and the aggregation of mitochondrial protein dihydrolipoamide S‐acetyltransferase (DLAT) due to fatty acylation, which induces cell cuproptosis. Additionally, it has been reported that STEAP4 (six‐transmembrane epithelial antigen of the prostate 4) in the membrane acts as a reductase and reduces Cu^2+^ to Cu^+^,^[^ ^37^, ^38^, ^39^ ^]^ which could be subsequently transported into cells by SLC31A1 (solute carrier family 31 member 1).^[^ ^38^, ^40^, ^41^ ^]^ Overexpression of STEAP4 increases intracellular reactive oxygen species (ROS) and is associated with the progression of prostate cancer.^[^ ^42^, ^43^ ^]^ However, whether STEAP4 might regulate cell cuproptosis is completely unknown. Moreover, it is also unknown whether Li exposure might cause cuproptosis to induce miscarriage.

Therefore, as far as we know, the association, causation, and underlying mechanism among Li exposure, cuproptosis, and miscarriage are completely uncovered. To explore these, we collected serum and villous tissue samples from a UM case‐control group (*n* = 50), constructed several LiCl‐exposed mouse models, and also made a LiCl‐exposed trophoblast Swan 71 cell model. We find that Li exposure causes cuproptosis to induce miscarriage by promoting FOXO1 (Forkhead Box O1)‐mediated STEAP4 transcription. Taken together, this study not only reveals novel biological mechanisms and pathogenesis of Li exposure‐induced unexplained miscarriage, but also provides potential targets for treatment against miscarriage, giving a typical example of Li exposure in the energy, environment, and health field.

## 2 Results

### 2.1 Li Exposure Induced Miscarriage

#### 2.1.1 Li Internal Exposure Levels were Positively Associated with Unexplained Miscarriage

To explore the potential association between Li internal exposure and the incidence of unexplained miscarriage, we conducted a new case‐control study containing 50 unexplained miscarriage (UM) patients and 50 their matched healthy control (HC) group, excluding the known clinical causes of miscarriage. Some variables that were associated with miscarriage based on prior studies^[^ ^44^, ^45^, ^46^ ^]^ were also collected, such as baseline characteristics, clinical information, and lifestyle (Table S5, Supporting Information). These characteristics did not show significant differences between these two groups (Table S5, Supporting Information).

**Figure 1:**
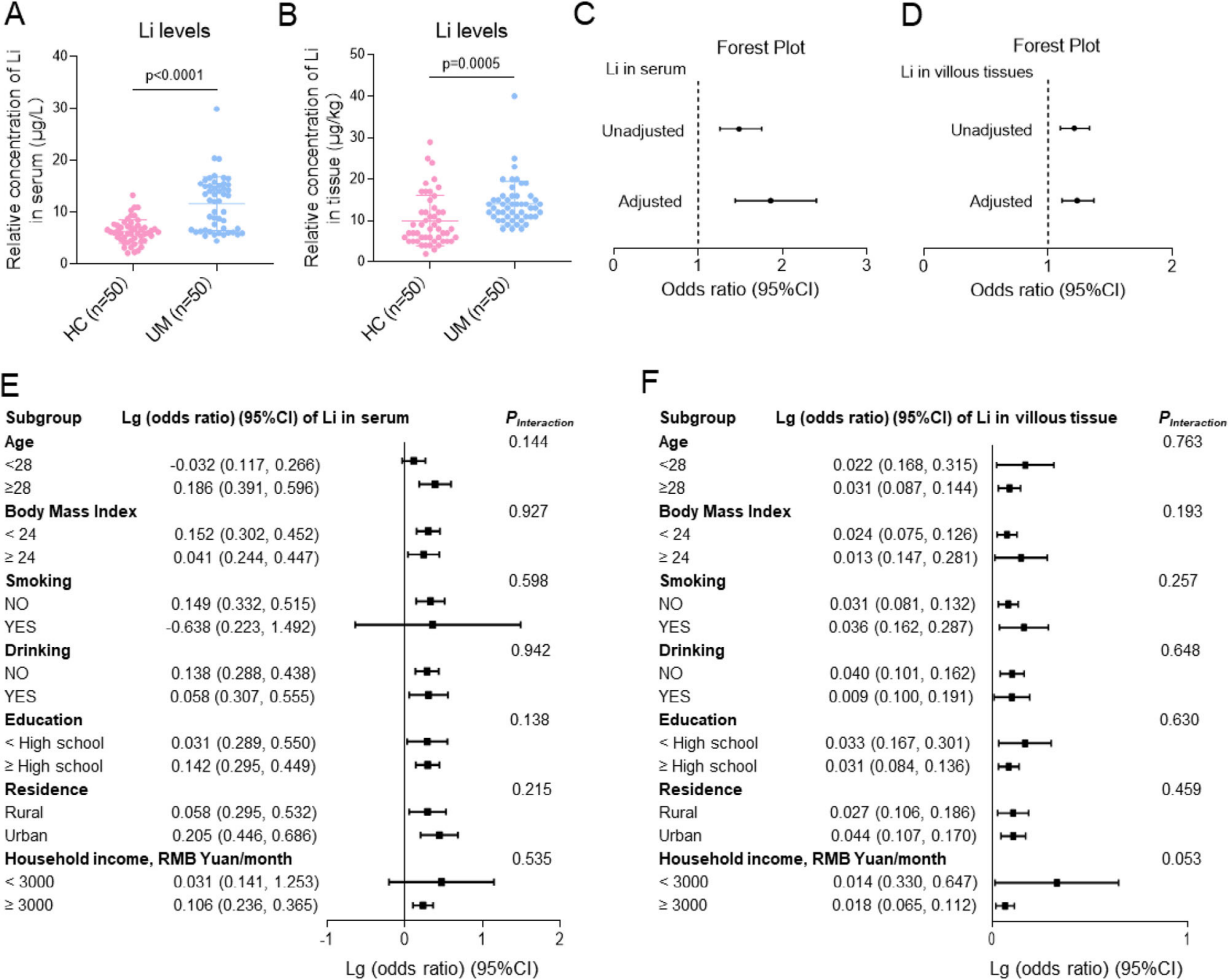
Li internal exposure levels were positively associated with unexplained miscarriage. A, B) The levels of lithium (Li, µg/L) in HC and UM (A) serum samples (*n = *50) and (B) villous tissues (*n = *50). C, D) Univariate and multivariate logistic regression analysis of the association of Li levels in serum samples (C) or Li levels in villous tissues (D) with unexplained UM. E, F) The associations between Li levels in serum samples (E) or villous tissues (F) and unexplained miscarriage by stratification analysis (all *p* for interaction > 0.05). HC, healthy control; UM, unexplained miscarriage.

Li levels were detected in HC and UM serum and villous tissue samples. Serum Li levels were significantly higher in the UM group (median (IQR) of 6.130 (4.680, 7.400)) than those in the HC group (median (IQR) of 12.26 (6.470, 15.18)) (**Figure** **1**A). Meanwhile, Li levels in villous tissues were also significantly higher in the UM group (mean ± SD of 9.740 ± 5.580 µg kg^−1^) than those in the UM group (mean ± SD of 14.60 ± 5.206 µg kg^−1^) (Figure 1B). In the unadjusted model, univariate logistic regression analysis showed that higher levels of Li in serum (OR = 1.486, 95% CI, 1.259–1.754) or in villous tissues (OR = 1.210, 95% CI, 1.099–1.333) were positively associated with unexplained miscarriage (Figure 1C). Based on directed acyclic graph analysis and previous literature,^[^ ^47^, ^48^ ^]^ age, BMI, education, household income, smoking, drinking, and residence were considered as potential confounders (Figure S1A, Supporting Information). To reduce their effects, we further analyzed the association between Li levels and miscarriage by adjusting for all these confounders, giving the adjusted OR value of 1.860 with 95% CI of 1.441–2.400 in serum samples and the adjusted OR value of 1.234 with 95% CI of 1.113–1.368 in villous tissue samples (Figure 1D; Table S6, Supporting Information). To further examine the association, stratification analysis showed that the stratifying factors did not significantly alter the association between Li levels and miscarriage (all *p* for interaction > 0.05, Figure 1E,F), Collectively, all the statistical models confirms that high levels of Li in serum or villous tissue samples were closely and positively associated with unexplained miscarriage.

#### 2.1.2 Li Exposure Induced Mouse Miscarriage

**Figure 2:**
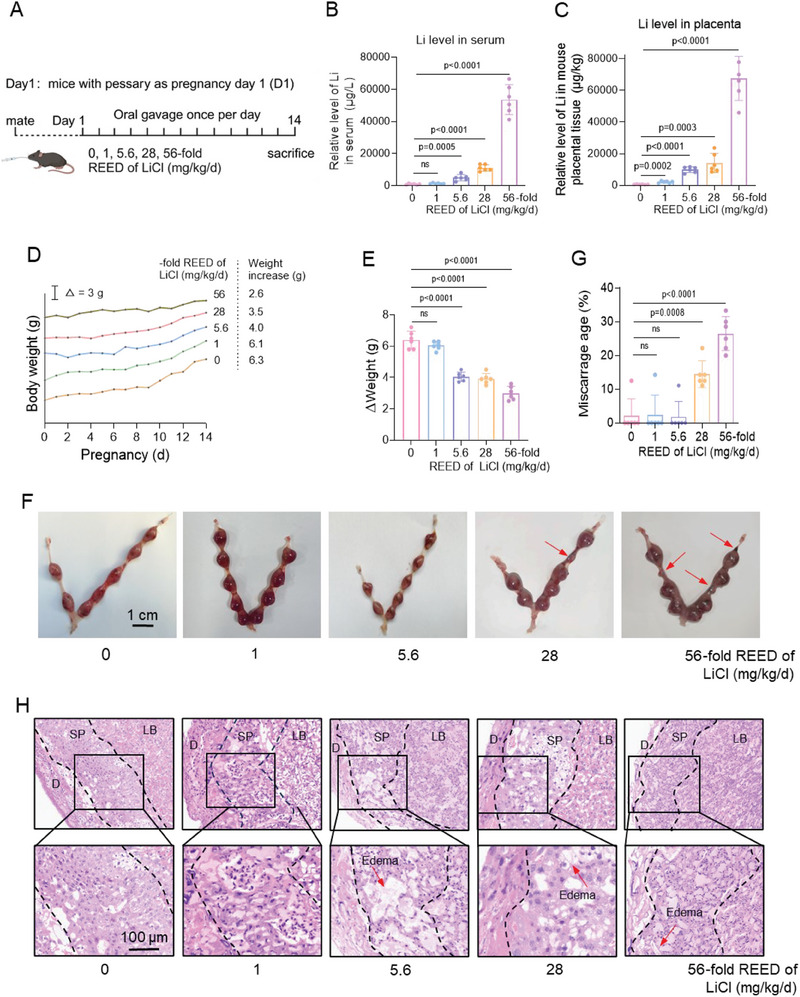
Li exposure induced mouse miscarriage. A) Schematic diagram of LiCl‐exposed mouse model. B, C) The levels of Li (µg/L) in mouse serum samples (*n = *6) and placental tissues (*n = *6). D) The increase in body weight of LiCl‐exposed pregnant mice with days. E) The increase in body weight of LiCl‐exposed pregnant mice on D14 relative to that on D1. F) Representative images of mouse embryo resorption (indicated by red arrows) in LiCl‐exposed mouse groups. G) The average miscarriage rates in LiCl‐exposed mouse groups. H) HE staining of placenta of LiCl‐exposed pregnant mice on Day 14. The decidua (D), spongiotrophoblast (SP), labyrinth (LB), and edema (E) were labeled (scale bar, 100 µm).

Subsequently, we explored the causality of whether Li exposure might induce miscarriage. To this end, we constructed a LiCl‐exposed mouse model, in which pregnant mice were treated with 0, 1‐fold, 5.6‐fold, 28‐fold, or 56‐fold REED (real environment exposure dose) of LiCl to mimic control, the real environmental exposure dose, median dose, therapeutic dose, or adverse outcome exposure dose, respectively (**Figure** **2**A). We found that Li exposure increased Li levels in serum and placental tissues (Figure 2B,C), lowered the rate of weight gain (Figure 2D,E), increased embryo resorption (Figure 2F), and elevated miscarriage rates (Figure 2G) in a dose‐dependent manner. In addition, HE staining also showed that the placenta became loose and produced edema in the sponge layer (spongiotrophoblast, SP) area after Li exposure compared with the control group (Figure 2H). Therefore, these results confirmed that Li exposure induced mouse miscarriage.

### 2.2 Li Exposure Induced Miscarriage by Causing Cuproptosis

#### 2.2.1 mRNA Sequencing Data Indicated that Li Exposure, Cuproptosis, and Miscarriage were Associated

**Figure 3:**
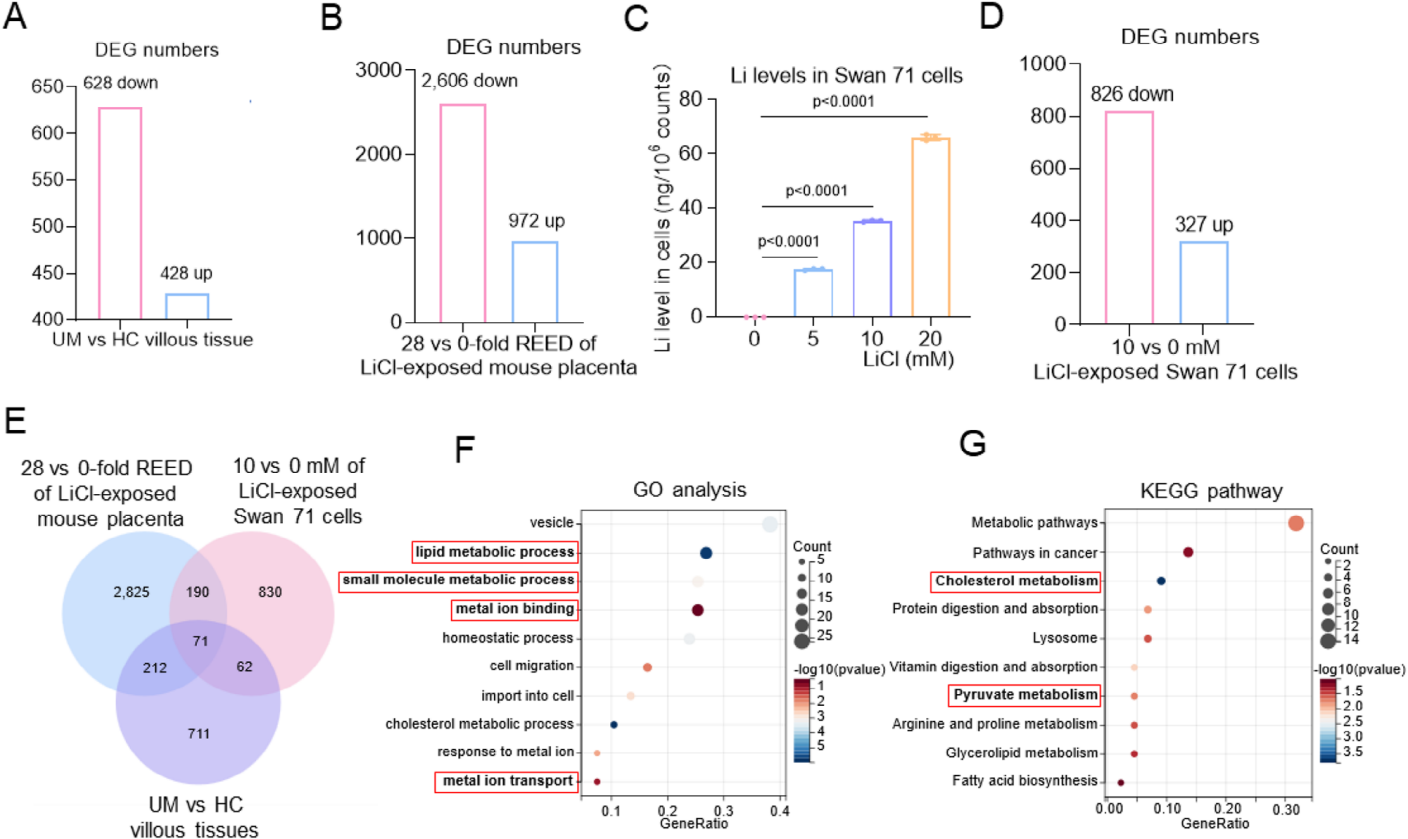
mRNA sequencing data demonstrated that Li exposure, cuproptosis, and miscarriage might be correlated. A, B) The number of differentially expressed genes (DEGs) in UM versus HC villous tissues and in 28 versus 0‐fold REED of LiCl‐exposed mouse placenta. C) Li levels in LiCl‐exposed Swan 71 cells. D) The DEGs in 10 versus 0 mM LiCl‐exposed Swan 71 cells. E) The intersection of three mRNA sequencing datasets by Venn diagram. F, G) GO and KEGG analysis of the DEGs in the intersection of (E).

Having known the association and causation between Li exposure and miscarriage, we further explored which cell phenotypes might be involved in Li exposure‐induced miscarriage. For this aim, three pairs of random HC and UM villous tissues were used for high‐throughput mRNA sequencing, giving 428 up‐regulated mRNAs and 628 down‐regulated mRNAs with a difference > 1.3‐fold and *p* < 0.05 (**Figure** **3**A). In mouse model, since 100 mg^−1^ kg^−1 ^d^−1^ LiCl (28‐fold of REED of Li) was sufficient to induce mouse miscarriage phenotype (Figure 2F), we selected 100 versus 0 mg^−1^ kg^−1 ^d^−1^ LiCl‐exposed mouse placental tissues for mRNA sequencing, giving 2606 up‐regulated and 972 down‐regulated mRNAs with difference > 1.5‐fold and *p* < 0.05 (Figure 3B). Trophoblast cells play important roles in healthy pregnancy, and Swan 71 cells have been widely used as a trophoblast cell model in miscarriage studies.^[^ ^49^ ^]^ Swan 71 cells were treated with 0, 5, 10, or 20 mM LiCl, and the intracellular Li levels were significantly increased after Li exposure (Figure 3C). Since 10 mM LiCl was sufficient to suppress cell viability (**Figure** **4**A), we selected 10 versus 0 mM LiCl‐exposed Swan 71 cells for high‐throughput mRNA sequencing, giving 826 up‐regulated and 327 down‐regulated mRNAs with differences > 1.5‐fold and *p* values < 0.05 (Figure 3D). In the intersection of three mRNA sequencing data, there were 71 changed (28 up‐regulated and 43 down‐regulated) mRNAs (Figure 3E). GO and KEGG analysis of these differentially expressed mRNAs (43+28 = 71) showed that small molecule metabolic process, metal ion binding, metal ion transport, and lipid metabolic process were the top significantly regulated (Figure 3F,G). Cuproptosis was mainly due to the suppression of mitochondrial respiration, the lipoacylation of proteins such as DLAT, and the reduction of iron‐sulfur cluster proteins. In GO and KEGG analysis (Figure 3F,G), pyruvate was regarded as the upstream of mitochondrial respiration, and its metabolism was closely related to mitochondrial respiration. Fatty acids were the raw materials of lipoic acid, and lipid metabolism was closely related to lipoacylation. The deficiency of small molecules such as iron sulfide affected the biosynthesis of iron‐sulfur cluster proteins, and the accumulation of ROS could lead to damage to iron‐sulfur cluster proteins. Together with the enrichment of metal ion transport, all these entries implied that cuproptosis might be top‐regulated. Therefore, these results demonstrated that Li exposure, cuproptosis, and miscarriage might be closely associated.

Figure 4Trophoblast cell, villous tissue, and mouse model assays confirmed that Li exposure caused cuproptosis to induce miscarriage. CCK8 assay analysis of cell viability of 0, 0.02, 5, 10, or 20 mM LiCl‐exposed Swan 71 cells for 0, 12, 24, 36, or 48 h. B) CCK8 assay analysis of cell viability of 10 mM LiCl‐exposed Swan 71 cells treated with various cell death inhibitors, such as TTM, ES, Fer‐1, Nec‐1, Z‐VAD‐FMK, 3‐MA, or TCEP, for 0, 12, 24, 36, or 48 h. C) The relative levels of Cu^+^ ions in 0, 0.02, 5, 10, or 20 mM LiCl‐exposed Swan 71 cells, as detected with CS1 as fluorescent probe. D, E) Western blot analysis of the protein levels of LIAS, SLC31A1, FDX1, and oligo‐DLAT in LiCl‐exposed Swan 71 cells and their relative quantification. F, G) The relative levels of Cu^+^ ions in HC and UM villous tissues, as detected with CS1 as fluorescent probe. H, I) Western blot analysis of the protein levels of LIAS, SLC31A1, FDX1, and oligo‐DLAT in HC and UM villous tissues and their relative quantification. J) The relative levels of Cu^+^ ions in 0, 5.6, 28, or 56‐fold REED of LiCl‐exposed mouse placenta. K, L) Western blot analysis of the protein levels of murine Lias, Slc31a1, Fdx1, and Oligo‐Dlat in LiCl‐exposed mouse placenta and their relative quantification. M) Schematic diagram of LiCl‐exposed mouse model with TTM treatment. Pregnant mice (each *n* = 6) were treated with saline or 56‐fold REED of LiCl by oral gavage and also with DMSO or TTM (10 mg kg^−1^ 3d^−1^) by intraperitoneal injection for continuous 13 day. (N‐O) Western blot analysis of the protein levels of murine Lias, Slc31a1, Fdx1, and Oligo‐Dlat in placental tissues of 56‐fold REED of LiCl‐exposed mice with TTM supplement (each *n* = 6) and their relative quantification. (P‐Q) Embryo resorption (indicated by red arrows) and the average miscarriage rates in 56‐fold REED of LiCl‐exposed mice with TTM supplement (each *n* = 66).

**Figure:**
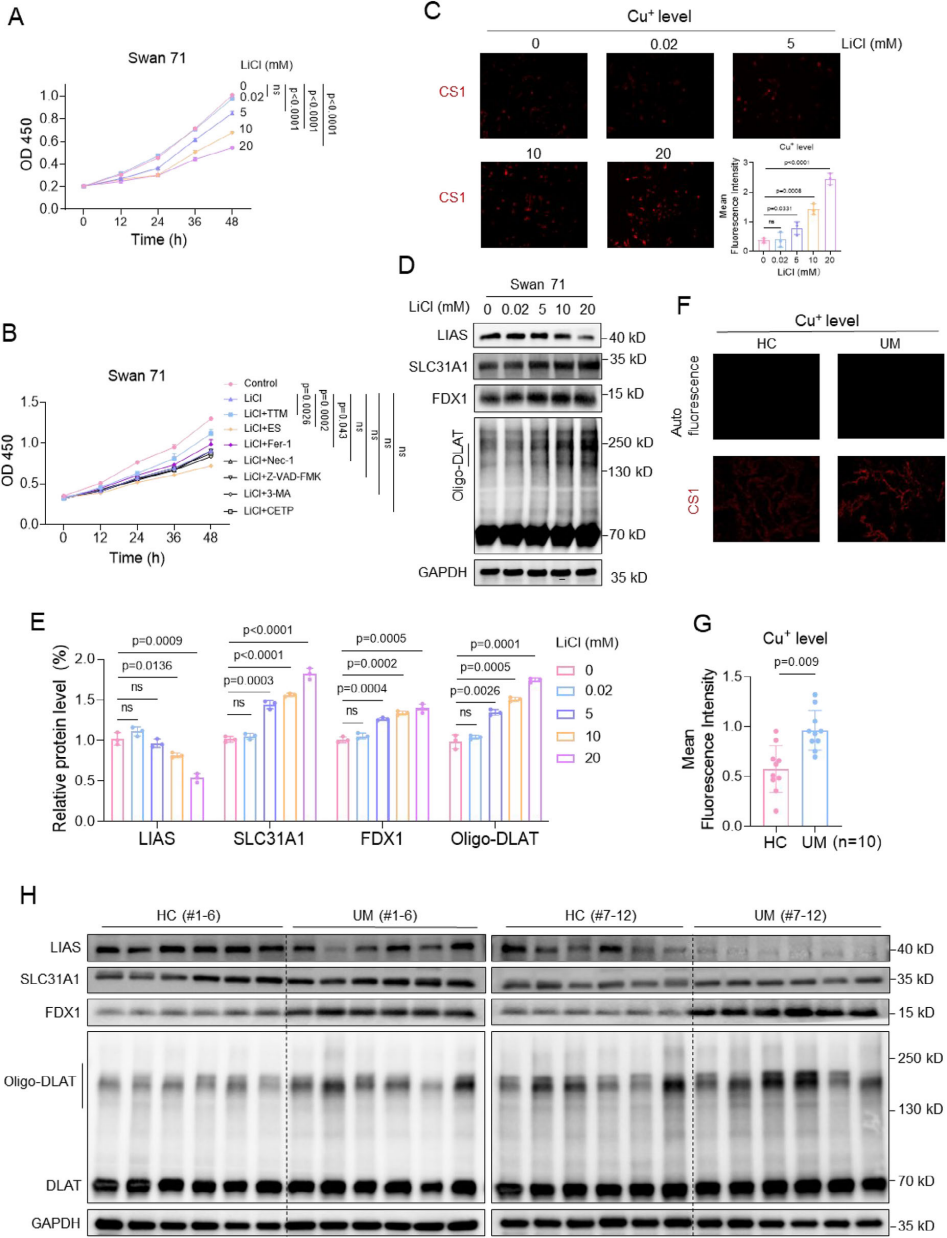

**Figure:**
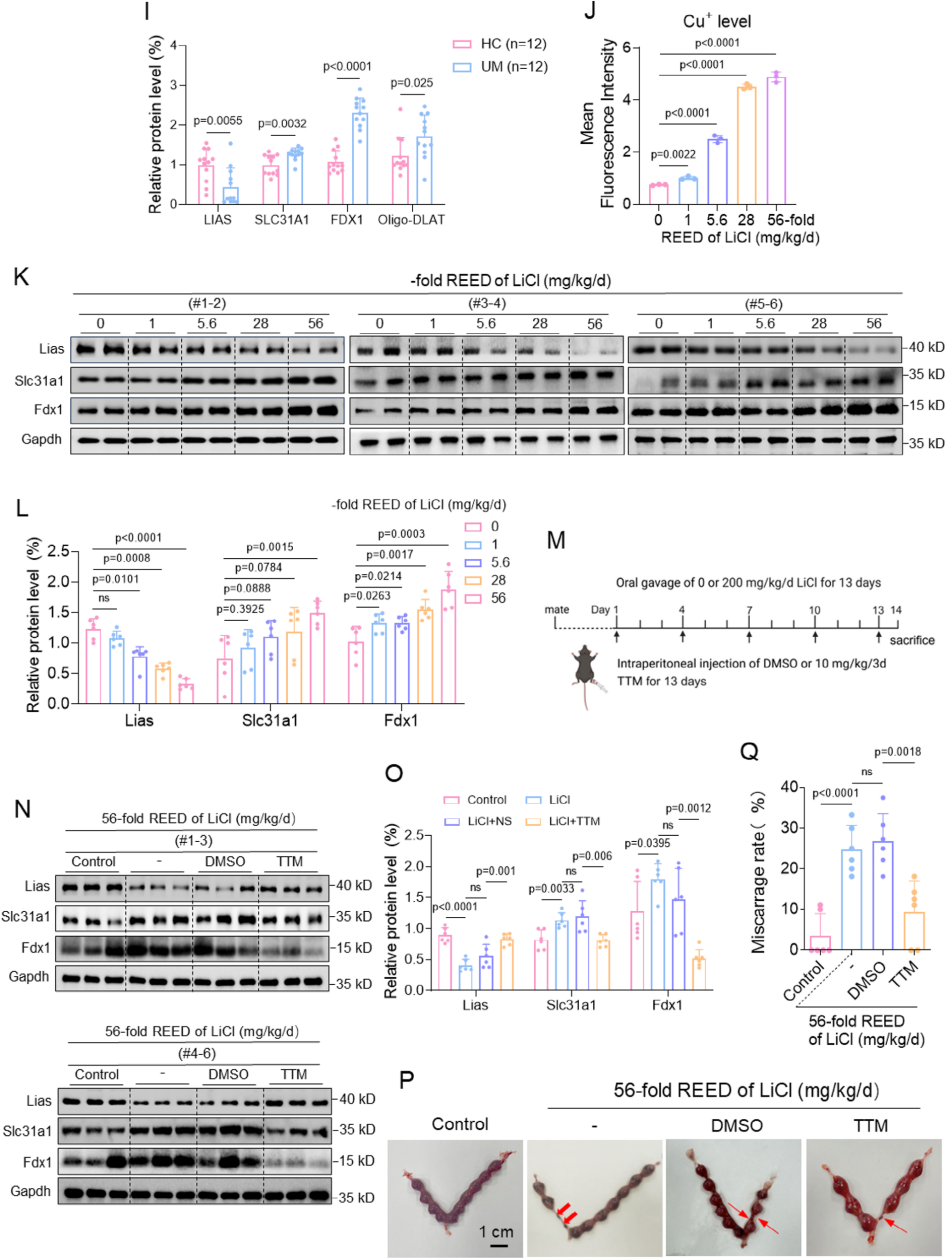

#### 2.2.2 Trophoblast Cell, Villous Tissue, and Mouse Model Assays Confirmed that Li Exposure Caused Cuproptosis to Induce Miscarriage

To experimentally confirm this, we detected the levels of several indicators of cuproptosis in LiCl‐exposed trophoblast cells, HC and UM villous tissues, and LiCl‐exposed mouse placental tissues. Among them, cell viability could be regarded as a good indicator to evaluate cell toxicity. Li exposure reduced Swan 71 cell viability (Figure 4A). In 10 mM LiCl‐exposed Swan 71 cells, co‐treatment with ES (a typical promoter of cuproptosis) further suppressed cell viability. However, co‐treatment with TTM (a typical inhibitor of cuproptosis) or Fer‐1 (an inhibitor of ferroptosis) could, whereas co‐treatment with Nec‐1 (necroptosis inhibitor), Z‐VAD‐FMK (apoptosis inhibitor), 3‐MA (autophagy inhibitor), or TCEP (disulfidptosis inhibitor) could not, restore the cell viability (Figure 4B, Figure S4I, Supporting Information). Notably, among all these inhibitors, TTM showed the top recovery efficiency, indicating that Li exposure primarily caused Swan 71 cell cuproptosis. Second, Li exposure also increased the levels of intracellular free Cu^+^ ions (Figure 4C), increased the protein levels of SLC31A1 (Cu^+^ importation protein), oligo‐DLAT (the oligomerization of lipoylated DLAT), and FDX1 (promote proteolipid acylation), and reduced the protein levels of LIAS (lipoic acid synthetase) in a dose‐dependent manner (Figure 4D,E). These results confirmed that Li exposure indeed caused Swan 71 cuproptosis. ATP7B (ATPase copper transport β) transports copper ions outside cells. However, the mRNA sequencing data did not show a significant difference in ATP7B mRNA levels (Figure S2A, Supporting Information). Experimental assays also confirmed that LiCl exposure did not affect the mRNA and protein levels of ATP7B in trophoblast cells (Figure S2B–D, Supporting Information). Taken together, these results supported that Li exposure caused trophoblast cell cuproptosis.

The levels of cuproptosis indicators were also detected in UM and HC villous tissues. The levels of Cu^+^ ions were higher in UM versus HC villous tissues (Figure 4F,G). The protein levels of SLC31A1, FDX1, and oligo‐DLAT were higher and those of LIAS were lower in UM versus HC villous tissues (Figure 4H,I). Multivariate logistic regression analysis by adjusting for all these confounders showed that the protein levels of LIAS, FDX1, and SLC31A1 were associated with unexplained miscarriage (Figure S3A, Supporting Information). Therefore, the levels of cuproptosis were higher in UM versus HC villous tissues, and higher levels of cuproptosis in villous tissues were positively associated with unexplained miscarriage. Moreover, the levels of Li in villous tissues were positively correlated with the protein levels of FDX1 or SLC31A1 but negatively with those of LIAS in UM villous tissues (Figure S3C–E, Supporting Information). The datapoints in the HC and UM groups were relatively separated. Collectively, these results confirmed that Li internal exposure, cuproptosis in villous tissues, and unexplained miscarriage were positively correlated with each other.

The levels of cuproptosis indicators were also detected in LiCl‐exposed mouse placental tissues. First, the levels of Cu^+^ ions were higher in LiCl‐exposed placenta tissues in a dose‐dependent manner (Figure 4J; Figure S3F, Supporting Information). The mRNA and amino acid sequences of SLC31A1, FDX1, DLAT, and LIAS were all conserved in rhesus, mouse, dog, and elephant (Table S8, Supporting Information). The protein levels of murine Slc31a1, Fdx1, Lias, and oligo‐Dlat were higher, and those of Lias were lower in LiCl‐exposed mouse placental tissues (Figure 4K–L). To further explore whether Li exposure induced miscarriage through placental cuproptosis, we reduced the levels of placental cuproptosis in LiCl‐exposed mice by intraperitoneal injection with TTM (a typical inhibitor of cuproptosis) (Figure 4M). TTM treatment recovered the protein levels of murine Slc31a1, Fdx1, Lias, and oligo‐Dlat in LiCl‐exposed mouse placental tissues (Figure 4N,O). Furthermore, TTM treatment also reduced embryo resorption and miscarriage rates (Figure 4P,Q). These results indicated that the reduction in placental cuproptosis levels could efficiently reduce the miscarriage rate in this Li‐exposed mouse model. Taken together, these results confirmed that Li exposure induced mouse miscarriage by causing placental cuproptosis in this Li‐exposed mouse model.

### 2.3 Underlying Mechanism of how Li Exposure Caused Cuproptosis

#### 2.3.1 Li Exposure Caused Trophoblast Cell Cuproptosis by Up‐Regulating STEAP4

Subsequently, we explored which molecule might be involved in Li exposure‐caused trophoblast cell cuproptosis. In the intersection (126 genes) in Figure 3E, we selected the top 20 up‐regulated and top 20 down‐regulated genes in all three datasets and re‐analyzed three of the total 40 genes in a Venn diagram (**Figure** **5**A). In the intersection, we identified that APOC1, STEAP4, and HSD11B1 were the top significantly differentially expressed in all three datasets (Figure S4A, Supporting Information). To validate them, using trophoblast cells, we found that Li exposure significantly up‐regulated the mRNA and protein levels of STEAP4, but not APOC1 and HSD11B1, in a dose‐dependent manner (Figure 5B,C; Figure S4B–E, Supporting Information), demonstrating that STEAP4 might be involved in Li exposure‐caused cuproptosis and Li exposure‐induced miscarriage. To further investigate the regulatory roles of STEAP4 in trophoblast cell cuproptosis, we constructed STEAP4‐overexpressing or ‐silenced trophoblast cells. Overexpression of STEAP4 reduced, whereas knockdown of STEAP4 increased, trophoblast cell viability (Figure 5D). Overexpression of STEAP4 increased, whereas knockdown of STEAP4 decreased, the levels of intracellular Cu^+^ ions in trophoblast cells (Figure 5E). Overexpression of STEAP4 also up‐regulated the protein levels of SCL31A1, FDX1, and oligo‐DLAT but down‐regulated those of LIAS; whereas knockdown of STEAP4 gave the opposite results (Figure 5F–I; Figure S4F, Supporting Information). Moreover, in STEAP4‐overexpressing Swan 71 cells, co‐treatment with ES further suppressed cell viability (Figure 5J). However, co‐treatment with TTM or Fer‐1 could, whereas co‐treatment with Nec‐1, Z‐VAD‐FMK, 3‐MA, or TCEP could not, effectively recover cell viability (Figure 5J; Figure S4H, Supporting Information). Notably, among all these inhibitors, TTM showed the top recovery efficiency. All these results confirmed that STEAP4 caused trophoblast cell cuproptosis. In LiCl‐exposed trophoblast cells, co‐knockdown of STEAP4 recovered the levels of cuproptosis indicators by increasing trophoblast cell viability (Figure 5K), reducing the levels of intracellular Cu^+^ ions (Figure 5L,M), up‐regulating the protein levels of SCL31A1, FDX1, and oligo‐DLAT, and down‐regulating the protein levels of LIAS (Figure 5N,O). These results indicated that LiCl exposure caused trophoblast cell cuproptosis by up‐regulating STEAP4 expression levels, and knockdown of STEAP4 could effectively suppress Li exposure‐caused trophoblast cell cuproptosis.

Figure 5Li exposure caused trophoblast cell cuproptosis by up‐regulating STEAP4. A) Re‐analysis of 40 top‐regulated (20 up‐regulated and 20 down‐regulated) DEGs in UM versus HC villous tissues, 28 versus 0‐fold REED of LiCl‐exposed mouse placenta, and 10 versus 0 mM LiCl‐exposed Swan 71 cells (total 126 genes) by Venn diagram. B) RT‐qPCR analysis of STEAP4 mRNA levels in LiCl‐exposed Swan 71 cells (*n = *3). C) Western blot analysis of STAEP4 protein levels in LiCl‐exposed Swan 71 cells and its relative quantification. D) CCK8 assay analysis of cell viability in LiCl‐exposed Swan 71 cells with overexpression or knockdown of STEAP4 for 0, 12, 24, 36, or 48 h. E) The relative levels of Cu^+^ ions in Swan 71 cells with overexpression or knockdown of STEAP4, as detected with CS1 as fluorescent probe (*n* = 66). F–I) The protein levels of STEAP4, LIAS, SLC31A1, FDX1, and Oligo‐DLAT in Swan 71 cells with knockdown or overexpression of STEAP4 and their relative quantification. J) CCK8 assay analysis of Swan71 cell viability with overexpression of STEAP4 and with co‐treatment with TTM, ES, Fer‐1, Nec‐1, Z‐VAD‐FMK, 3‐MA, or TCEP for 0, 12, 24, 36, or 48 h. K) CCK8 assay analysis of cell viability of 10 mM LiCl‐exposed Swan 71 cells with knockdown of STEAP4 for 0, 12, 24, 36, or 48 h. L, M) The relative levels of Cu^+^ ions in 10 mM LiCl‐exposed Swan 71 cells with knockdown of STEAP4, as detected with CS1 as fluorescent probe (*n* = 66). (N‐O) Western blot analysis of the protein levels of STEAP4, LIAS, SLC31A1, FDX1, and Oligo‐DLAT in 10 mM LiCl‐exposed Swan 71 cells with knockdown of STEAP4 and their relative quantification.

**Figure:**
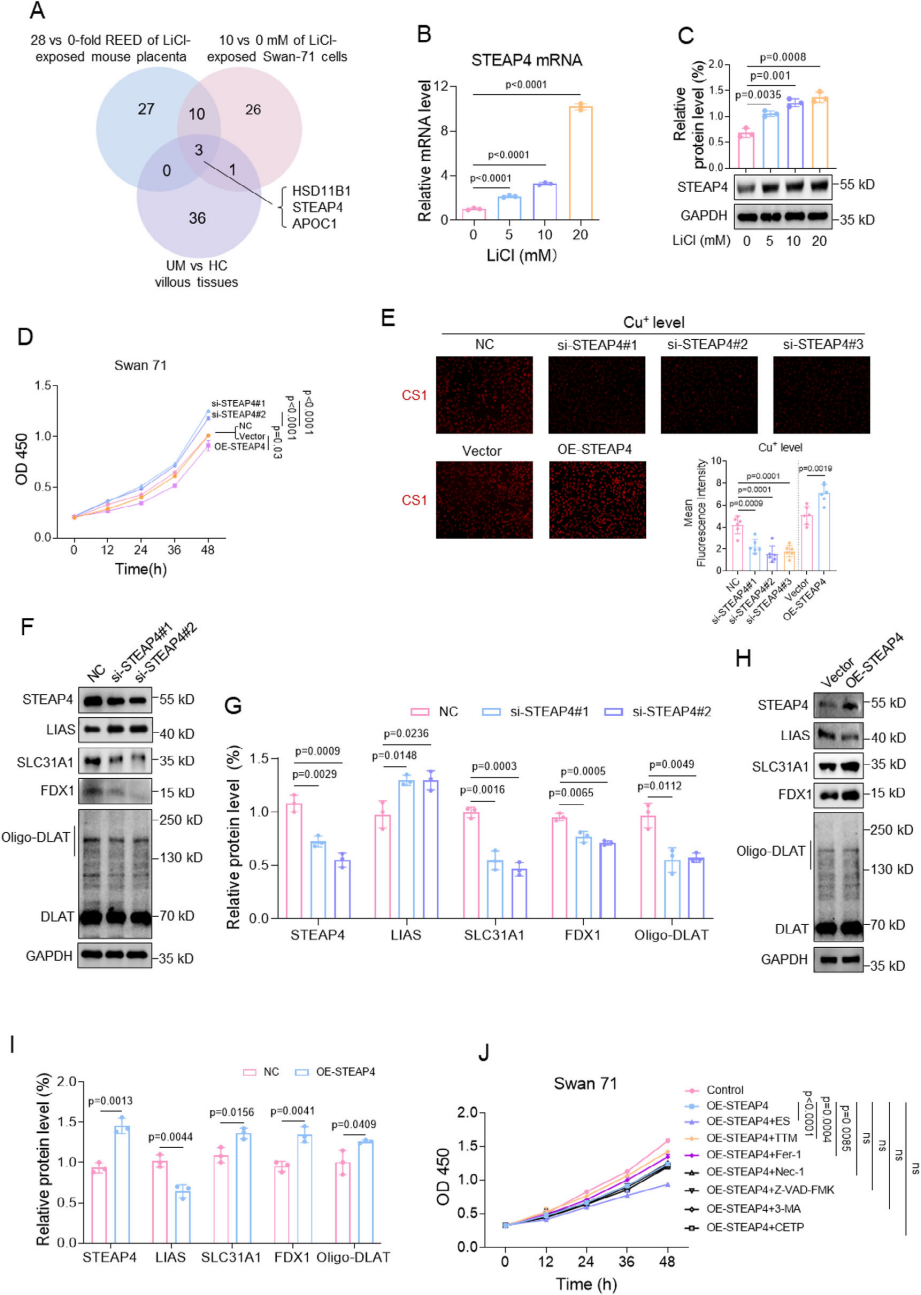

**Figure:**
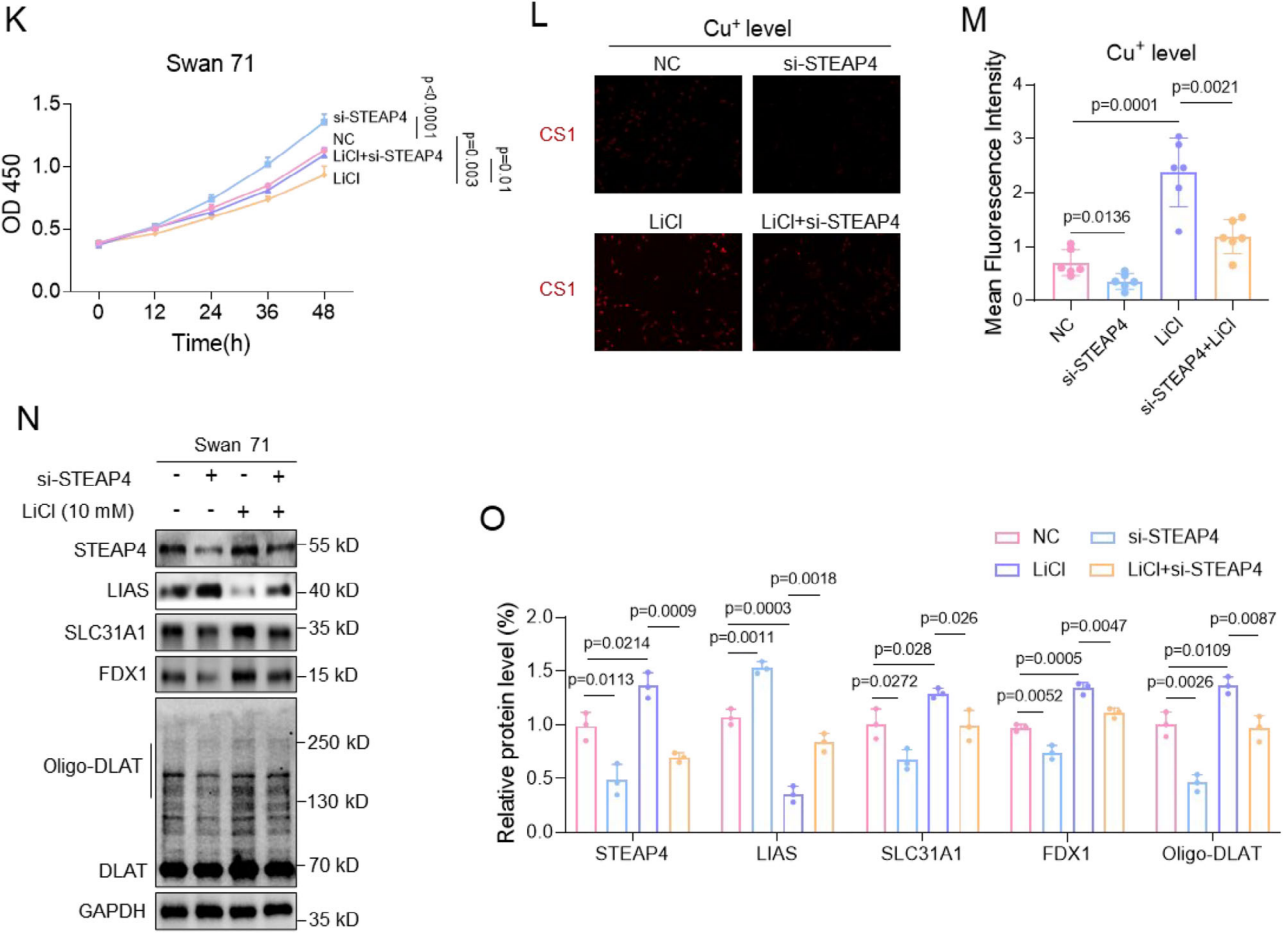

#### 2.3.2 LiCl Exposure Promoted FOXO1‐Mediated STEAP4 Transcription

Next, we investigated how Li exposure up‐regulated the mRNA levels of STEAP4. Li exposure up‐regulated the mRNA levels of STEAP4 (Figure 5C) but did not affect its mRNA stability (Figure S5A, Supporting Information), suggesting that Li exposure might promote STEAP4 mRNA transcription. To identify its potential transcription factor, FOXO1 was predicted as a transcription factor of STEAP4 as analyzed by JASPAR, PROMO, and STITCH, three widely used software for the prediction of transcription factors (**Figure** **6**A). Experimentally, Li exposure up‐regulated the mRNA and protein levels of FOXO1 in human trophoblast cells (Figure 6B; Figure S5B, Supporting Information). Overexpression of FOXO1 up‐regulated, whereas knockdown of FOXO1 down‐regulated, the mRNA and protein levels of STEAP4 in human trophoblast cells (Figure 6C; Figure S5C–F, Supporting Information). FOXO1 ChIP assays showed that the promoter region of STEAP4 could be enriched by FOXO1 in trophoblast cells, and the enriched levels were further increased with Li exposure (Figure 6D). Furthermore, luciferase assays discovered that FOXO1 showed transcription activity using the wild‐type (wt), but not mutant (mut), promoter region of STEAP4 in trophoblast cells, and the transcription activity was further increased after Li exposure (Figure 6E). Therefore, these results confirmed that FOXO1 was the transcription factor of STEAP4, and Li exposure promoted FOXO1‐mediated STEAP4 transcription. In LiCl‐exposed trophoblast cells, Li exposure up‐regulated the mRNA and protein levels of FOXO1 and STEAP4; and co‐knockdown of FOXO1 down‐regulated their expression levels (Figure 6F,G; Figure S5G,H, Supporting Information). Moreover, Li exposure caused trophoblast cell cuproptosis by up‐regulating the protein levels of LIAS, SLC31A1, and FDX1; and co‐knockdown of FOXO1 suppressed trophoblast cell cuproptosis (Figure 6H,I). Therefore, these results showed that Li exposure caused trophoblast cell cuproptosis by up‐regulating FOXO1 expression levels and by promoting FOXO1‐mediated STEAP4 transcription in trophoblast cells.

Figure 6Li exposure promoted FOXO1‐mediated STEAP4 transcription. A) Venn diagram analysis of the transcription factors of STEAP4 analyzed by JASPAR, STITCH, and PROMO. B) FOXO1 protein levels in LiCl‐exposed Swan 71 cells and its relative quantification. C) STEAP4 protein levels in Swan 71 cells with overexpression of FOXO1 and its relative quantification. D) FOXO1 ChIP assay analysis of the levels of STEAP4 promoter region enriched by FOXO1 in 10 mM LiCl‐exposed Swan 71 cells. E) Dual‐luciferase reporter assay analysis of the transcription activity of FOXO1 using wild‐type (Wt) or mutant (Mut) promoter sequence of STEAP4 in 10 mM LiCl‐exposed Swan 71 cells. F–G) The protein levels of FOXO1 and STEAP4 in 10 mM LiCl‐exposed Swan 71 cells with knockdown of FOXO1 and their relative quantification. (H‐I) The protein levels of LIAS, SLC31A1, and FDX1 in 10 mM LiCl‐exposed Swan 71 cells with knockdown of FOXO1 and their relative quantification. J, K) The levels of the remained FOXO1 protein in 10 mM LiCl‐exposed Swan 71 cells and with CHX (10 µM) treatment for 0–8 h and its relative quantification. L) The root mean square deviation (RMSD) analysis of the protein structural stability of FOXO1 (UniProt ID: Q12778) exposed to 10 mM LiCl (with NaCl as control) by molecular dynamics (MD) simulations using GROMACS within 100 ns. M–O) The 3D protein structure of 10 mM LiCl (with NaCl as control)‐exposed FOXO1 by MD simulations using GROMACS within 100 ns, as visualized by PyMOL v2.5.4. The 2D structures of FOXO1 were analyzed using PDBsum. NaCl‐exposed FOXO1 contained α‐helices (111 amino acid residues in red), β‐sheets (16 residues in yellow), and random coils (528 residues in green). LiCl‐exposed FOXO1 contained α‐helices (139 residues), β‐sheets (22 residues), and random coils (494 residues). (P) The binding energy of LiCl‐exposed or NaCl‐exposed (as control group) FOXO1‐STEAP4 promoter region was analyzed by gmx_MMPBSA v1.6.1 (single trajectory method).

**Figure:**
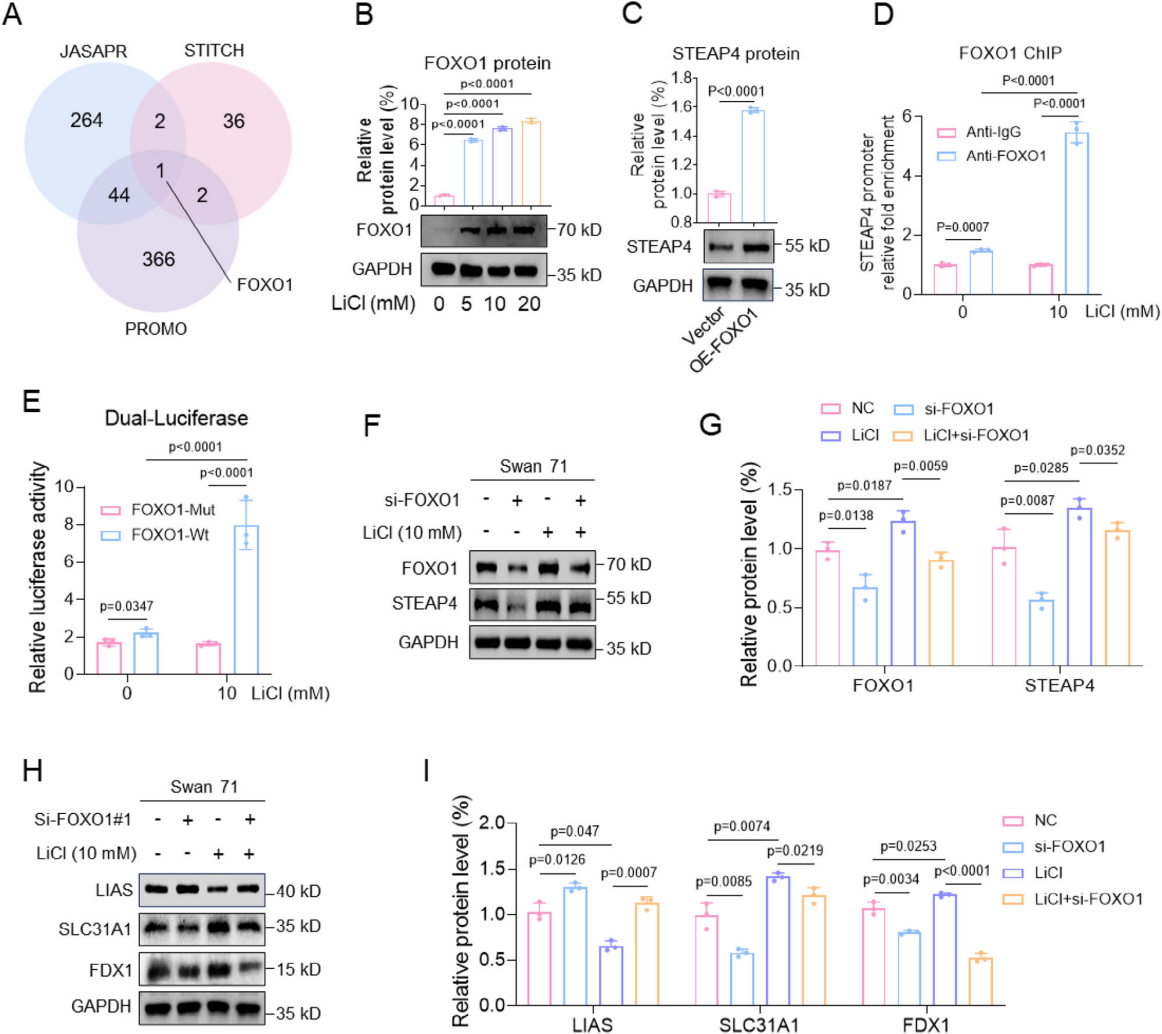

**Figure:**
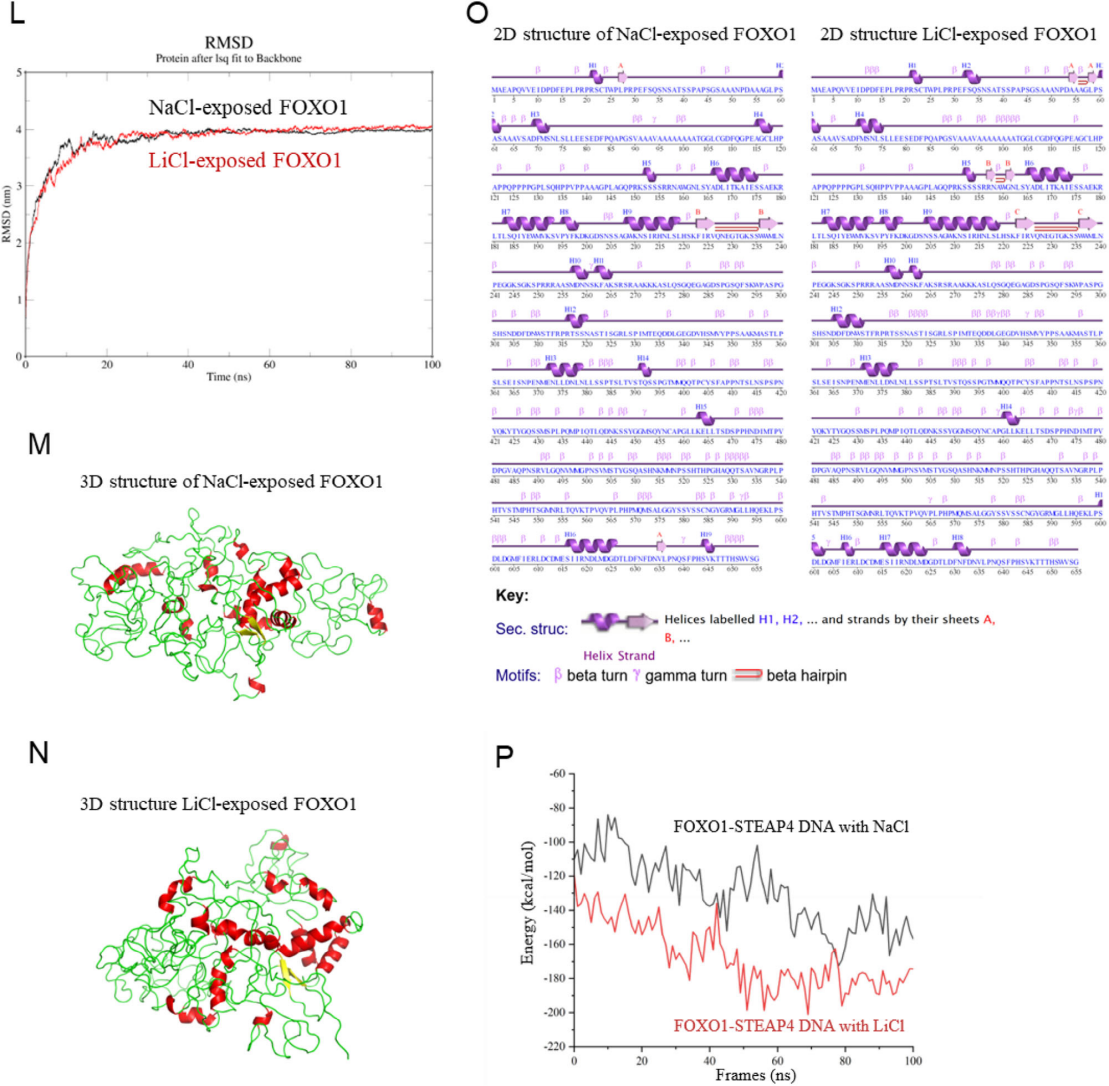

Subsequently, we further explored whether Li might affect FOXO1 protein structure. To identify this, we analyzed FOXO1 protein structure by molecular simulation. The 3D and 2D structures of FOXO1 (UniProt ID: Q12778) were constructed by the AlphaFold database (Figure S5I,J, Supporting Information). The Ramachandran plot showed the strong overall quality of FOXO1 protein 3D structure (Figure S5K, Supporting Information). Then, molecular dynamics (MD) simulations of LiCl‐exposed FOXO1 (with NaCl‐exposed FOXO1 as control) showed that the values of root mean square deviation (RMSD) of Li‐exposed FOXO1 were similar to (or slightly lower in 0–20 ns than) that in the control protein (Figure 6L). Structurally, LiCl‐exposed FOXO1 increased the number of amino acid residues in α‐helices (139 vs 111) and that in β‐sheets (22 vs 16) and reduced that in random coils (494 vs 528) relative to NaCl‐exposed FOXO1 as control (Figure 6M–O), indicating that Li exposure might stabilize the ordered architecture of FOXO1 protein. Collectively, these results demonstrated that Li exposure might increase the structural stability of FOXO1 protein, which might explain why Li exposure suppressed FOXO1 protein degradation.

Subsequently, we further investigated the effects of Li exposure on the binding between FOXO1 protein and STEAP4 promoter region by MD simulations. The complex structures of DNA‐binding region (residues 151–249) in FOXO1 and STEAP4 promoter region (5′‐TGAAAGACATGGGGTCTGACCAACC‐3′) were predicted by AlphaFold3. The FOXO1‐STEAP4 promoter complex contained 4 hydrophobic interactions, 14 H‐bonds, 1 salt bridge, and 1 π‐Stacking interaction **(Figure** **S5** **L**, Supporting Information), supporting that FOXO1 could bind with the STEAP4 promoter region. Then, we compared the structural difference between the LiCl‐exposed FOXO1‐STEAP4 promoter region and the NaCl‐exposed FOXO1‐STEAP4 promoter region as a control group. Compared with the control group, the Li‐exposed FOXO1‐STEAP4 promoter region had more H‐bonds (25 vs 23), fewer salt bridges (6 vs 7), and more hydrophobic interactions (4 vs 2) (Figure S5M,N, Supporting Information). The average binding energy of Li‐exposed FOXO1‐STEAP4 promoter region was −167.19 kcal mol^−1^, lower than −129.77 kcal mol^−1^ for the control group (Figure 6P; Table S9, Supporting Information), indicating that Li exposure increased the binding between FOXO1 and STEAP4 promoter region, which might explain that Li exposure promoted FOXO1‐mediated STEAP4 transcription.

#### 2.3.3 FOXO1 and STEAP4 were Highly Expressed in UM Versus HC Villous Tissues and in Li‐Exposed Mouse Placental Tissues

To validate the cellular mechanisms in villous tissues, we detected the expression levels FOXO1 and STEAP4 in both UM and HC villous tissues. The mRNA and protein levels of FOXO1 and STEAP4 were significantly higher in UM versus HC villous tissues (**Figure** **7**A–D). FOXO1 ChIP assays showed that the levels of the promoter region of STEAP4 enriched by FOXO1 were higher in UM versus HC villous tissues (Figure 7E). Multivariate logistic regression analysis by adjusting for all these confounders showed that the protein levels of FOXO1 and STEAP4 were associated with unexplained miscarriage (Figure S3B, Supporting Information). Pearson correlation analysis showed that Li levels were positively correlated with the protein levels of FOXO1 and STEAP4 in UM villous tissues (Figure 7F,G). The protein levels of FOXO1 and STEAP4 were also positively correlated in UM villous tissues (Figure 7H). The datapoints in both UM and HC groups were obviously separated (Figure 7F–H). Combined with the cellular results, we proposed that Li exposure might upregulate this FOXO1/STEAP4 axis and thus increase the levels of cuproptosis in UM versus HC villous tissues, which further induced miscarriage.

Figure 7FOXO1 and STEAP4 were highly expressed in UM versus HC villous tissues and in Li‐exposed mouse placental tissues. A, B) The mRNA levels of FOXO1 and STEAP4 in HC and UM villous tissues (each *n = *12). C, D) The protein levels of FOXO1 and STEAP in HC and UM villous tissues and their relative quantification (each *n = *12). E) FOXO1 ChIP assay analysis of the levels of STEAP4 promoter region enriched by FOXO1 in HC and UM villous tissues (*n = *6). F–H) Pearson correlation analysis of the correlation among Li levels in serum, FOXO1 protein levels in villous tissues, and STEAP4 protein levels in villous tissues in HC and UM groups (each *n = *12). I–J) ROC curves of the mRNA levels of murine Foxo1 and Steap4 in LiCl‐exposed mouse placental tissues (each *n = *6). (K‐L) The protein levels of Foxo1 and Steap4 in LiCl‐exposed mouse placental tissues (each *n = *6) and their relative quantification. M) Foxo1 ChIP assay analysis of the levels of Steap4 promoter region enriched by Foxo1 in LiCl‐exposed mouse placental tissues (each *n = *6). (N‐P) Pearson correlation analysis of the correlation among Li levels, Foxo1 protein levels, and Steap4 protein levels in LiCl‐exposed mouse placenta (each *n = *6).

**Figure:**
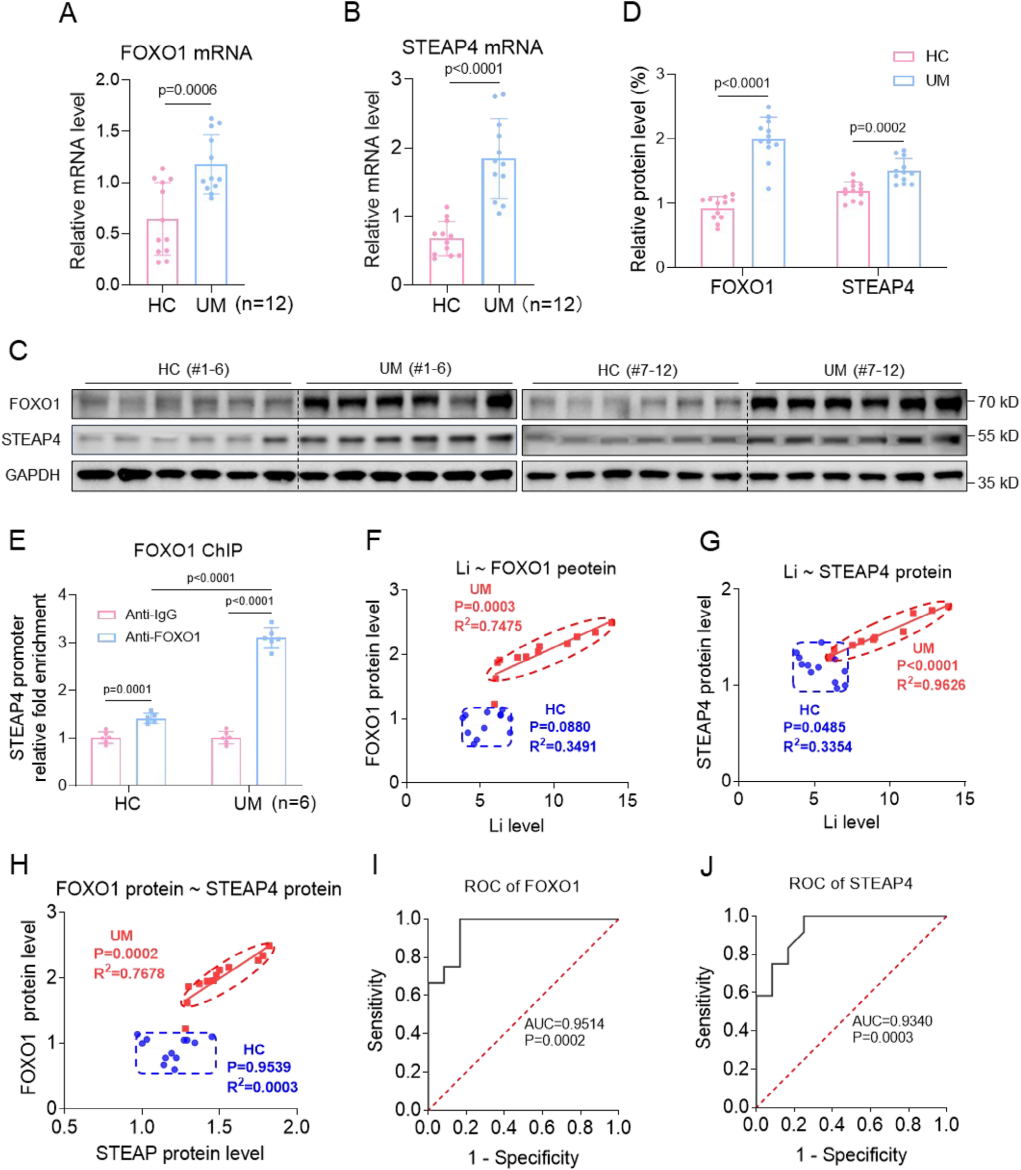

**Figure:**
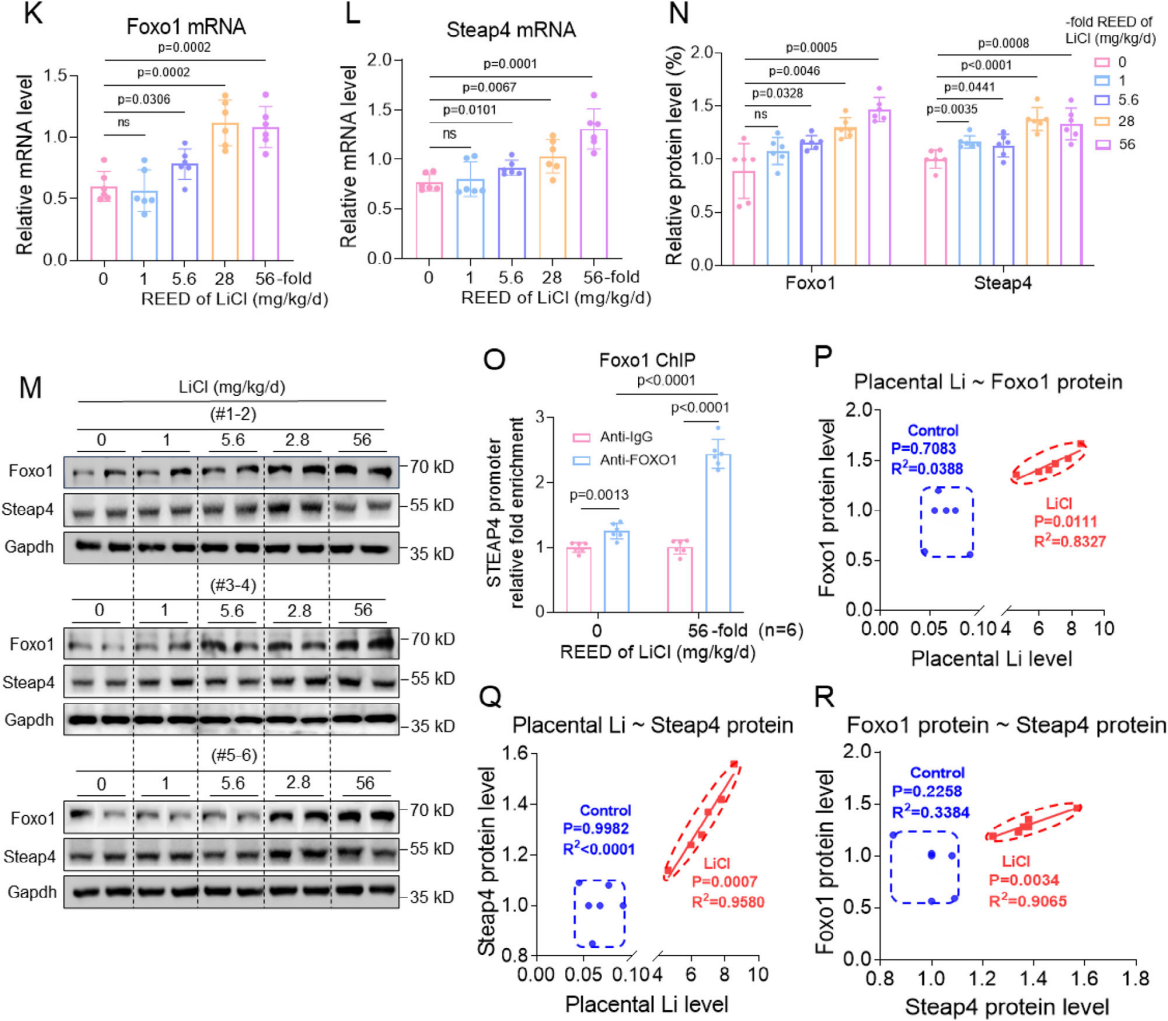

The expression levels of murine Foxo1 and Steap4 were also detected in LiCl‐exposed mouse placental tissues. First, the mRNA and amino acid sequences of FOXO1 and STEAP4 were all conserved in human, rhesus, mouse, dog, and elephant (Table S8, Supporting Information). The mRNA and protein levels of murine Foxo1 and Steap4 were higher in LiCl‐exposed mouse placental tissues (Figure 7K–N). Foxo1 ChIP assays showed that the levels of the promoter region of Steap4 enriched by Foxo1 were higher in LiCl‐exposed mouse placental tissues (Figure 7O). Pearson correlation analysis showed that Li levels were positively correlated with the protein levels of Foxo1 or Steap4 in Li‐exposed mouse placental tissues (Figure 7P,Q). The protein levels of Foxo1 and Steap4 were also positively correlated in Li‐exposed mouse placental tissues (Figure 7R). Combined with the cellular results, we proposed that Li exposure might upregulate this FOXO1/STEAP4 axis and thus cause cuproptosis in placental tissues and further induce miscarriage in this LiCl‐exposed mouse model.

### 2.4 Treatment Against Li Exposure‐Induced Miscarriage

#### 2.4.1 Miscarriage Treatment by Therapeutic Down‐Regulating Foxo1 or Steap4 in LiCl‐Exposed Mouse Placental Tissues

Finally, we explored how to treat a miscarriage that was induced by Li exposure. To this end, we constructed a miscarriage treatment model in which LiCl‐exposed pregnant mice were intraperitoneally injected with AS1842856 (a typical suppressor of FOXO1) or Forskolin (a typical suppressor of STEAP4) to examine its effects on placental cuproptosis and mouse miscarriage rates (Figure S6A,B, Supporting Information). As validation, treatment with AS1842856 or Forskolin down‐regulated the protein expression levels of Foxo1 or Steap4 in mouse trophoblast cells, respectively (Figure S6C,D, Supporting Information). This treatment also down‐regulated Foxo1 or Steap4 protein levels in LiCl‐exposed mouse placental tissues (**Figure** **8**A–D). As a result, this treatment restored (i.e., decreased) the levels of Cu^+^ ions and (i.e., increased) the protein levels of Slc31a1, Lias, and Fdx1 in placental tissues of LiCl‐exposed mice (Figure 8E–J). As for phenotype, this treatment reduced embryo resorption and miscarriage rates (Figure 8K–N). Collectively, these results showed that therapeutic down‐regulation of Foxo1 or Steap4 could efficiently reduce placental cuproptosis and mouse miscarriage rates in this Li‐exposed mouse model.

Figure 8Miscarriage treatment by therapeutic down‐regulating Foxo1 or Steap4 in LiCl‐exposed mouse placenta. A–D) The protein levels of murine Foxo1 and Steap in placental tissues of 56‐fold REED of LiCl‐exposed mice with AS1842856 or Forskolin treatment (each *n = *6) and their relative quantification. E, F) The relative levels of Cu^+^ ions in 56‐fold REED of LiCl‐exposed mouse placenta with AS1842856 or Forskolin treatment and its relative quantification (each *n = *6). G–J) The protein levels of Lias, Slc31a1, and Fdx1 in placental tissues of 56‐fold REED of LiCl‐exposed mice with AS1842856 or Forskolin treatment (each *n = *6) and their relative quantification. (K‐N) Embryo resorption (indicated by red arrows) and the average miscarriage rates in 56‐fold REED of LiCl‐exposed mice with with AS1842856 or Forskolin treatment (each *n = *6).

**Figure:**
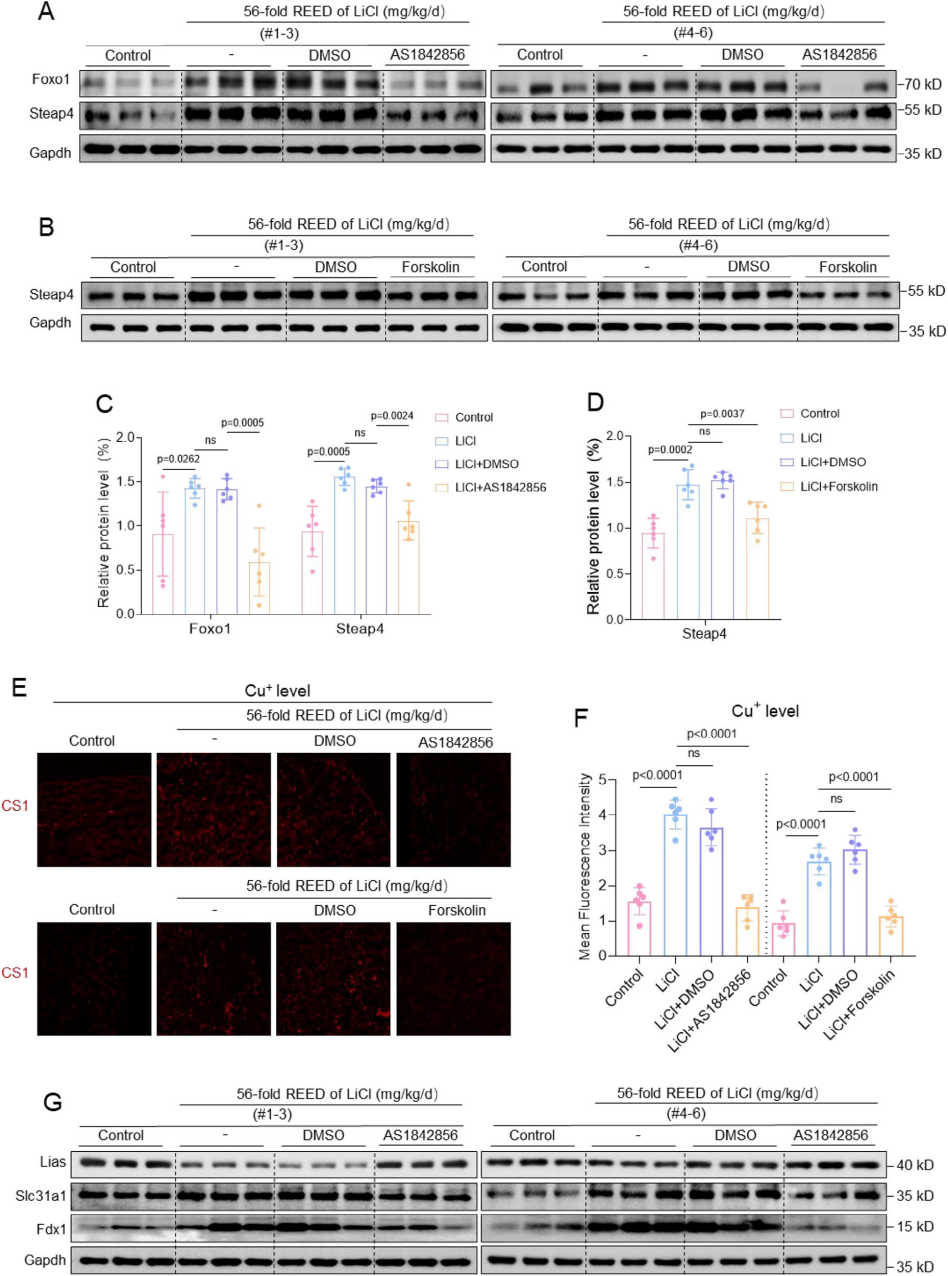

**Figure:**
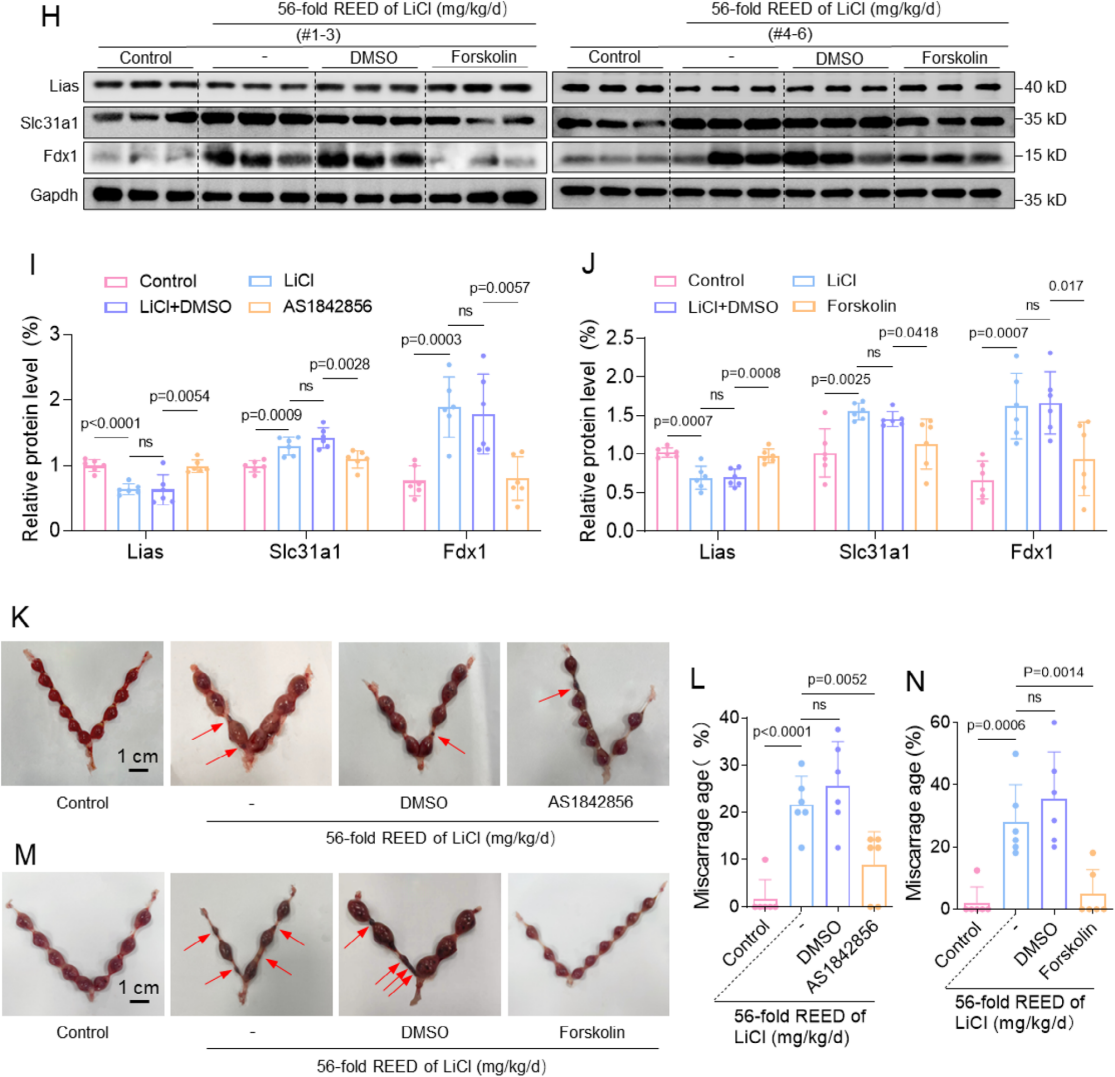

## 3 Discussion

### 3.1 Li Exposure Induces Miscarriage

**Figure 9:**
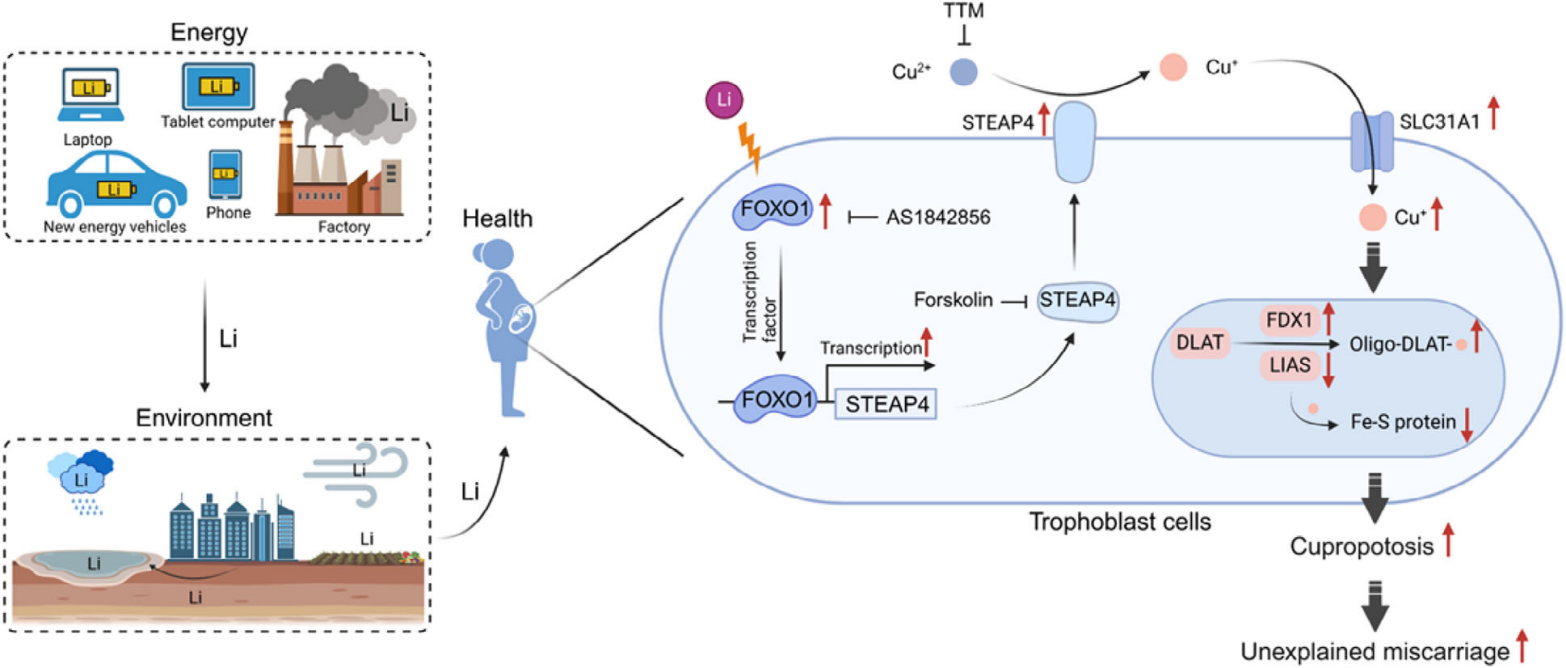
The proposed mechanisms that Li exposure causes cuproptosis to induce miscarriage. Li in various discarded Li batteries is widely spread into our environments and then transported into human bodies. Li exposure promotes FOXO1‐mediated STEAP4 transcription, increases the levels of Cu^+^ ions, promotes cuproptosis by up‐regulating SLC31A1, FDX1, and oligo‐DLAT and by down‐regulating LIAS, which ultimately induces unexplained miscarriage. Repression of mouse placental cuproptosis could efficiently alleviate mouse miscarriage.

With widely global application of Li batteries, environmental Li pollution and health problems are becoming important concerns in the energy‐environment‐health field. Li batteries have been widely used worldwide, but few are recycled, and thus, waste Li has been widely spread into the environment and eventually transported into the human body (**Figure** **9**). Epidemiological studies have shown that Li exposure during early pregnancy is associated with an increased risk of adverse pregnancy outcomes, such as major fetal malformations^[^ ^50^, ^51^ ^]^ and miscarriage.^[^ ^31^ ^]^ Animal model assays also show that exposure to LiCl and Li_2_CO_3_ can inhibit the growth and reproduction of *Caenorhabditis* elegans.^[^ ^52^ ^]^ However, whether Li exposure during pregnancy might induce unexplained miscarriage remains largely unknown. In this study, we find that Li levels in serum or villous tissues are significantly higher in UM versus the HC group, and the higher levels of Li are positively associated with unexplained miscarriage. Moreover, in the mouse model, we confirm that Li exposure induces mouse miscarriage. Therefore, it is the first time that we confirm that Li exposure induces unexplained miscarriage based on the comprehensive epidemiological analysis, mouse assays, and cellular mechanistic study, discovering a new risk factor for unexplained miscarriage and also revealing new important findings in the energy‐environment‐health field.

### 3.2 Li Exposure Induces Miscarriage by Causing Cuproptosis

How does Li exposure induce miscarriage? We find that Li exposure causes cuproptosis to induce unexplained miscarriage. Cuproptosis is a newly identified and Cu‐dependent cell death form,^[^ ^36^ ^]^ which is associated with the occurrence and progression of various diseases, such as the prognosis of tumors by altering CDKN2A expression levels,^[^ ^53^ ^]^ the death of spermatogenic cells in male mice,^[^ ^54^ ^]^ the arrest of follicular development in mouse granulosa cells,^[^ ^55^ ^]^ the progression of atherosclerosis and stroke progression,^[^ ^56^ ^]^ chronic obstructive pulmonary disease,^[^ ^57^ ^]^ the development of neurological dysfunction,^[^ ^58^ ^]^ and the development of Parkinson's disease.^[^ ^59^ ^]^ By mRNA sequencing and experimental validation, we identify that the levels of cuproptosis are higher in UM versus HC villous tissues, in placental tissues of LiCl‐exposed mice with miscarriage, and also in LiCl‐exposed human trophoblast cells. These results showed that Li exposure causes cuproptosis to induce miscarriage (Figure 9). Suppression of mouse placental cuproptosis levels by treating with TTM, AS1842856, or Forskolin could efficiently reduce LiCl‐exposed mouse miscarriage rates. In our recent studies, we have also found that copper exposure causes placental cuproptosis to induce miscarriage^[^ ^14^ ^]^; BaP/BPDE exposure leads to trophoblast cell cuproptosis by up‐regulating lnc‐HZ11^1^. Moreover, BaP/BPDE exposure also inhibits trophoblast cell migration/invasion, suppresses homologous recombination repair, or induces ferroptosis or pyroptosis, any of which might induce unexplained miscarriage.^[^ ^1^, ^11^, ^15^, ^60^ ^]^ Nanoplastics inhibit trophoblast cell migration/invasion and migrasome formation or lead to trophoblast cell apoptosis, which induces miscarriage.^[^ ^12^, ^13^ ^]^ Hypoxia causes ferroptosis to induce miscarriage.^[^ ^8^ ^]^ Disinfection by‐products in tap water alter the levels of hormones and further induce miscarriage.^[^ ^61^ ^]^ Collectively, all these environmental toxicants might induce unexplained miscarriage through multiple cellular death or dysfunctions, among which several toxicants (Cu, BaP/BPDE, or Li) induce cuproptosis, showing a complicated regulatory network of environmental toxicants on unexplained miscarriage.

In addition to cuproptosis, Li exposure also promotes apoptosis and exhibits anti‐tumor activity in pancreatic cancer cells or activates AKT/mTOR signaling to induce necroptosis in schwannoma tumor cells.^[^ ^62^, ^63^ ^]^ Li treatment also leads to pyroptosis in renal cells by activating NLPR3/caspase 3/6/7/9^[^ ^64^ ^]^ or causes ferroptosis in melanoma.^[^ ^65^ ^]^ Li exposure is also related to autophagy, with some inconsistent conclusions. For example, Li induces autophagy and promotes tumor cell death in skin melanoma^[^ ^66^ ^]^ but suppresses over‐activated autophagy by activating PI3K/AKT/mTOR signaling in rats.^[^ ^67^ ^]^ Therefore, Li exposure regulates multiple cell phenotypes. Other kinds of cytotoxicity should be further explored.

### 3.3 Li Promotes FOXO1‐Mediated STEAP4 Transcription to Cause Trophoblast Cell Cuproptosis

The underlying mechanisms by which Li exposure causes trophoblast cell cuproptosis are proposed (Figure 9). First, Li up‐regulates the expression levels of FOXO1, which acts as a transcription factor of STEAP4 to promote its transcription (Figure 6J). STEAP4 reduces Cu^2+^ to Cu^+[^ ^39^ ^]^; and the up‐regulated STEAP4 increased the levels of Cu^+^, which is subsequently transported into trophoblast cells through SLC31A1. High levels of intracellular Cu^+^ ions promote cuproptosis by up‐regulating SLC31A1, FDX1, and oligo‐DLAT, and by down‐regulating LIAS. Furthermore, trophoblast cell cuproptosis ultimately induces unexplained miscarriage. The cellular mechanisms are consistent with those in UM villous tissues and in LiCl‐exposed mouse placental tissues. Repression of mouse placental cuproptosis by treating with TTM or therapeutic down‐regulation of FOXO1 with AS1842856 or down‐regulation of STEAP4 with Forskolin could reduce placental cuproptosis to alleviate miscarriage in the LiCl‐exposed mouse models. It has been reported that the elevated FOXO1 level is a poor prognostic indicator for epithelial ovarian cancer.^[^ ^68^, ^69^ ^]^ Meanwhile, FOXO1 also functions as a cell‐specific transcription factor that is involved in endometrial remodeling during early pregnancy, which is closely related to female reproductive health.^[^ ^70^ ^]^ The low expression levels of STEAP4 are associated with obesity and play a crucial role in regulating insulin sensitivity in human adipocytes,^[^ ^68^, ^69^ ^]^ and its high expression levels in human prostate are associated with prostatitis.^[^ ^71^ ^]^ Herein, we find that the high expression levels of FOXO1 or STEAP4 in the placenta are involved in LiCl‐exposed cuproptosis and miscarriage. Collectively, Li exposure promotes FOXO1‐mediated STEAP4 transcription, causes trophoblast cell cuproptosis, and induces miscarriage, providing novel pathways and essential mechanisms for Li exposure‐induced cuproptosis and miscarriage.

### 3.4 Miscarriage Intervention

Recently, increasing studies have uncovered the molecular mechanisms of unexplained miscarriage.^[^ ^1^, ^10^, ^47^, ^48^, ^72^, ^73^, ^74^, ^75^ ^]^ For example, fructose‐1,6‐diphosphate has been reported to prevent miscarriage by inducing COX‐2 macrophage differentiation in the decidua.^[^ ^75^ ^]^ Also, increasing succinate levels intravenously during the first trimester reduces the risk of recurrent spontaneous miscarriage.^[^ ^74^ ^]^ Progesterone therapy may improve pregnancy outcomes in women who have bleeding in early pregnancy.^[^ ^76^ ^]^ The anti‐progesterone drug (mifepristone and the prostaglandin misoprostol) can be used to treat a missed miscarriage.^[^ ^77^ ^]^ The glucocorticoid delivery system GC‐Exo‐CD16Ab effectively inhibits the cytotoxicity of decidual natural killer cells, improving placental and fetal morphology, and ultimately reducing the risk of mouse miscarriage.^[^ ^78^ ^]^ In our recent studies, we have found that supplementing with GPX4 could suppress ferroptosis, recover angiogenesis, and alleviate BaP‐induced mouse miscarriage.^[^ ^15^ ^]^ Knockdown of either murine lnc‐hz06 or Ncoa4 could efficiently suppress ferroptosis and alleviate miscarriage in hypoxic mouse model^[^ ^8^ ^]^; knockdown of murine Ahr or Ctip recovers homologous recombination repair in placental tissues and alleviates miscarriage in a BaP‐exposed mouse miscarriage model^[^ ^60^ ^]^; knockdown of Nlrp3 reduces placental pyroptosis and alleviates BaP‐induced mouse miscarriage^[^ ^10^ ^]^; knockdown of lnc‐Hz11 or lnc‐Hz06 recovers trophoblast cell migration/invasion and alleviate BaP‐induced mouse miscarriage^[^ ^15^ ^]^; supplement with murine Sox2 or Rock1 rescues migration/invasion and migrasome formation and alleviates polystyrene nanoplastics (PS‐NPs)‐induced miscarriage^[^ ^12^ ^]^; supplement with Bcl‐2 suppresses apoptosis in PS‐NPs‐exposed trophoblast cells and alleviates miscarriage in PS‐NPs‐exposed pregnant mouse model^[^ ^13^ ^]^; and treatment with TTM (a cuproptosis inhibitor) suppresses placental cuproptosis and alleviates miscarriage in a CuCl_2_‐exposed mouse model.^[^ ^14^ ^]^ In this study, logistic regression analysis showed that higher levels of Li in serum samples or higher protein levels of FOXO1 or STEAP4 in villous tissues are associated with unexplained miscarriage (Figure 1C; Figure S3B, Supporting Information). ROC curve analysis shows that Li levels in serum samples or FOXO1 or STEAP4 protein levels in villous tissues could well predict the risk of miscarriage (Figure 7I,J). Mouse models also confirm that treatment with TTM (a suppressor of cuproptosis), AS1842856 (a suppressor of FOXO1), or Forskolin (a suppressor of STEAP4) could reduce placental cuproptosis and alleviate miscarriage in the LiCl‐exposed mouse models, providing new targets for effective treatment against unexplained miscarriage. AS1842856 is a specific FOXO1 inhibitor to suppress FOXO1 trans‐activation in the mouse liver.^[^ ^79^, ^80^, ^81^, ^82^ ^]^ Forskolin is a plant‐derived diterpene with excellent immunomodulatory properties and could induce cAMP formation and autophagy. Forskolin reduces STEAP4 expression levels in both cellular and animal models.^[^ ^83^ ^]^ The roles of AS1842856 and Forskolin in miscarriage prediction and treatment, as well as their accuracy, convenience, safety, delivery, and metabolism, remain to be further investigated. As for Li, Li levels are higher in UM versus HC serum samples, showing a positive association with miscarriage, which provides a new indicator for clinical prevention of unexplained miscarriage. In addition, pregnant women should avoid exposure to high‐dose Li in life, such as staying away from Li industrial areas and using Li according to the doctor's advice. Moreover, detecting Li levels or other metal ion levels in serum should be a concern in annual routine physical examination. Collectively, these studies showed that different environmental toxicants induce unexplained miscarriage through specific and different approaches. We provide specific targets for treatment against specific environmental toxicant‐induced unexplained miscarriage, enriching the therapeutic approach for various unexplained miscarriages.

### 3.5 Limitations and Prospects

To recycle and reuse Li batteries and to reduce environmental Li exposure are important topics in the energy‐environment‐health field, which should be addressed by global governments and enterprises. The transport pathway and organ enrichment of Li in humans remain unclear and need to be explored urgently. In addition, Li exposure might also lead to other dysfunctions of human trophoblast cells and induce other adverse pregnancy outcomes. In this work, we collect serum and villous tissue samples from 50 pairs of UM and HC women. More women should be enrolled to give more accurate results. There might be recall bias for the confounder collection in a retrospective case‐control study. Our ICP‐MS results show that, in addition to Li, the levels of other metal elements such as Zn and Ni are also higher in UM versus HC serum samples (Figure S1B, Supporting Information). However, the levels of other metal elements such as Mn, Fe, Co, As, Cd, Pb, or Cu in serum did not show a significant difference between HC and UM groups. Notably, ICP‐MS results give the total levels of a metal element but cannot distinguish the different metal valence states. In this study, we focus on Li exposure and its association with unexplained miscarriage. Although the total levels of Cu element were unchanged, Li exposure increases the levels of Cu^+^ ions in cells and tissues, which further induces cuproptosis. In further studies, other metals and unexplained miscarriage should also be investigated. Moreover, these sequencing data and cell viability also suggest that ferroptosis might be regulated by Li exposure, which should also be further investigated. Li exposure might also cause other diseases, rather than female reproduction. Finally, the treatment against unexplained miscarriage should be further explored for clinical translation.

## 4 Conclusion

Based on the epidemiological statistical analysis, mouse model, and cellular assays, we conclude that Li exposure causes trophoblast cell cuproptosis to induce unexplained miscarriage. Mechanistically, Li exposure upregulates FOXO1 expression and thus promotes FOXO1‐mediated STEAP4 transcription, which further causes cuproptosis to induce miscarriage. The cellular mechanisms are consistent with those in UM villous tissues and in Li‐exposed mouse placental tissues. Treatment with TTM to suppress cuproptosis, with AS1842856 to down‐regulate FOXO1, or with Forskolin to down‐regulate STEAP4 expression levels could efficiently suppress placental cuproptosis and alleviate miscarriage in the Li‐exposed mouse model. Collectively, this study not only discovers novel pathogenesis of Li‐induced unexplained miscarriage, but also reveals biological targets for treatment against unexplained miscarriage. This study proposes an important Li exposure theme in the energy‐environment‐health field, which deserves critical attention by global governments, enterprises, and hospitals.

## 5 Experimental Section

### Chemicals

LiCl (99.99% purity) was purchased from Aladdin (7447‐41‐8). LiCl was dissolved in ddH_2_O to make 5 mM LiCl stock. AS1842856 (836620‐48‐5) and Forskolin (66575‐29‐9) were purchased from MCE. Necrostatin‐1 (Nec‐1, 4311‐88‐0), Ferrostatin‐1 (Fer‐1, 347174‐05‐4), Z‐VAD‐FMK (187389‐52‐2), 3‐Methyladenine (3‐MA, 5142‐23‐4), Elesclomol (ES, 488832‐69‐5), and Ammonium tetrathiomolybdate (TTM, 15060‐55‐6) were purchased from Abmole. TCEP (51805‐45‐9) was purchased from Beyotime.

### Cell Culture

Swan 71 cells, which were immortalized by human telomerase, were constructed by Gil Mor's group at Yale University^[^ ^49^ ^]^ and were received as gifts. Swan 71 cells were cultured in DMEM/F12 medium (GIBCO, Invitrogen) supplemented with 10% FBS (GIBCO) at 37 °C in a humidified atmosphere containing 5% CO_2_. Mouse placental trophoblast cells were purchased from Procell Life Science & Technology Co., Ltd (CP‐M144, Hubei, China). Mouse trophoblast cells were cultured in DMEM/F12 medium (Gibco, C11330500BT).

The doses of LiCl in LiCl‐exposed trophoblast cells were calculated and selected. Li levels have been detected as 0.274‐20.9 µM in human blood and 0.015–0.663 mM in human urine^[^ ^33^ ^]^ in the normal population under real environmental exposure and as 1–5 mM in body fluids of bipolar disorder patients with intake of Li_2_CO_3_ as medicine.^[^ ^84^, ^85^ ^]^ Meanwhile, treatment of human breast cancer cells (MCF‐7) with 50 or 100 mM LiCl caused cell apoptosis by regulating GSK‐3β, caspase‐2, Bax, and cleaved‐caspase‐7.^[^ ^85^ ^]^ Treatment with 30 mM LiCl up‐regulated GSK‐3β protein levels, increased DNA damage, and suppressed the proliferation of MCF‐7 and MDA‐MB‐231 breast cancer cells.^[^ ^86^ ^]^ Therefore, based on the doses used in literature and pre‐experiments, in cellular assays, Swan 71 cells were treated with 0, 0.02, 5, 10, or 20 mM LiCl for 48 h. The dose of 0.02 mM LiCl was close to the highest Li levels in human blood in the normal population.

### Cell Transfection

The empty vector pcDNA3.1 (Catalog No. V790‐20) was purchased from Thermo Fisher Scientific Company. cDNAs that were used for construction of overexpression plasmid of STEAP4 (pcDNA3.1‐STEAP4) and FOXO1 (pcDNA3.1‐FOXO1) were synthesized and constructed into pcDNA3.1 vector by Addgene. The corresponding mRNA sequences were obtained from the National Center for Biotechnology Information (NCBI) database (Gene Bank, Homo sapiens, GRCh38.p14; sequences in Table S1, Supporting Information). Si‐STEAP4, si‐FOXO1, and si‐NC (negative control) were customized by Thermo Fisher (sequences in Table S2, Supporting Information). Swan 71 cells (1 × 10^6^ cells/well) were transfected with 1 µg plasmids or 50 nM siRNAs in TurboFect transfection reagent (R0531, Thermo Scientific) for 24 h according to the manufacturer's protocols. The transfection efficiencies were validated by RT‐qPCR.

### Cell Viability

Cell viability was evaluated using Cell Counting Kit‐8 (CCK8, ab228554, Abcam, Cambridge, UK).^[^ ^87^ ^]^ Cells (5 × 10^3^ cells per well, five replicates for each group) were seeded in 96‐well plates for 8 h and then treated with varying concentrations (0, 0.02, 5, 10, 20 mM) LiCl for another 48 h. Afterward, 10 µL incubation reagent and 90 µL DMEM/F12 medium were added according to the manufacturer's protocols. The 96‐well plate was fully covered with tin foil to avoid light. Subsequently, the plate was incubated at 37 °C for 1 h. The absorbance was measured at 450 nm using a microplate reader (Bio‐Rad, Hercules, CA, USA) with cell culture medium as background. The proliferation of cells was expressed as the change in absorbance at 450 nm. All experiments were replicated thrice.

### High‐Throughput mRNA Sequencing and Data Processing

Swan 71 cells (5 × 10^6^ cells) treated with 10 mM LiCl, together with an equal number of unexposed cells as a control, were used for mRNA sequencing. Three pairs of random HC and UM villous tissues, as well as placental tissues of 100 versus 0 mg/kg/day LiCl‐exposed mice, were also used for mRNA sequencing. Bioinformatic analysis was performed using Omicsmart, a dynamic, real‐time, interactive online platform for data analysis (http://www.omicsmart.com). Briefly, total RNAs were extracted by Trizol reagent (Thermo Fisher Scientific). The process included the removal of rRNA, synthesis of double‐stranded cDNA, end repair, degradation of one strand, and enrichment of the other strand by quantitative reverse transcription PCR (RT‐qPCR). The library quality was confirmed by sequencing. The differentially expressed genes (DEGs) with differences > 1.3‐fold (woman villous tissues) or 1.5‐fold (mouse placenta tissues and cells) and *p* < 0.05 were generated from read counts using the online bioinformatic platform Dr. Tom provided by BGI (biosys.bgi.com). These DEGs were searched in the NCBI database (Gene Bank, Homo sapiens, GRCh38.p14 for human and Gene Bank, Mus musculus, GRCm39 for mouse) to determine their genome loci. In the intersection of three mRNA sequencing datasets, the up‐regulated and down‐regulated mRNAs were combined and used for gene ontology (GO) and KEGG analysis to generate GO and KEGG plots, respectively.^[^ ^88^, ^89^ ^]^

### Quantitative Reverse Transcription PCR (RT‐qPCR)

Total RNAs were extracted from cells or tissues using Trizol (Invitrogen, Carlsbad, USA).^[^ ^90^, ^91^ ^]^ RNA quality and quantity were assessed using a NanoDrop 2000 UV spectrophotometer (Thermo Fischer Scientific, Waltham, USA) (Meyer‐Cifuentes et al. 2020). The RNA purity was high, as determined by the A260/280 values in the range of 1.8–2.0. The isolated RNAs (800 ng) were converted into cDNAs using the EasyScript All‐in‐One First‐Strand cDNA Synthesis SuperMix kit for cDNA synthesis, which contained gDNA Remover to remove the genomic DNA (the template). Then, cDNAs were amplified using a 20‐µL SYBR Green Supermix (Takara, Kyoto, Japan). The RT‐qPCR program was described as follows: pre‐denaturation at 95 °C for 30 s, cycling reaction at 95 °C for 10 s for 40 cycles, dissolution at 95 °C for 15 s, 60 °C for 60 s, and then 95 °C for 15 s. The sequences of the specific primers are shown in Table S3 (Supporting Information). The amplification results were automatically analyzed using 2^‐ΔΔCt^ method with CFX96TM Real‐time PCR (Bio‐Rad, CA, USA). GAPDH mRNA was used as an internal standard for all mRNAs. All experiments were replicated thrice. The levels of mRNAs were expressed as 2^‐ΔΔCt^, where Ct was the cycle threshold, ΔCt = testing gene (Ct) – average GAPDH (Ct), and ΔΔCt = sample group Δ(Ct) – average control group Δ(Ct).

### RNA Stability Analysis

Swan 71 cells (1 × 10^6^ cells/well) treated with LiCl were seeded in a 6‐well plate for 24 h. Then, the cells were treated with 5 µg mL^−1^ actinomycin D (Sigma‐Aldrich) to block mRNA transcription. After 0, 1, 2, 3, or 4 h, RNAs were extracted from cells, and the STEAP4 mRNA was analyzed by RT‐qPCR assays. GAPDH mRNA was used as the normalization internal standard.

### Western Blot Analysis

Total proteins were extracted using RIPA lysis buffer (Thermo Fisher Scientific) and quantified using Pierce BCA Protein Assay Kit (Pierce). Proteins (10–30 µg well^−1^, equal amounts within group but different amounts among groups for better comparison) were separated on 6%–12% SDS‐PAGE gel and transferred to an equilibrated polyvinylidene difluoride membrane (PVDF, Amersham Biosciences, Buckinghamshire, UK). After blocking with 5% bovine serum albumin (BSA, Sigma‐Aldrich) in TBST (10 mM Tris‐HCl, 150 mM NaCl, and 0.1% Tween 20) for 1 h at room temperature, the membrane was incubated with primary antibody overnight at 4 °C. The primary antibodies contained anti‐STEAP4 (861150, Zenbio, dilution 1:1000), anti‐FOXO1 (18592‐1‐AP, Proteintech, dilution 1:2000), anti‐SLC31A1 (R27288, Zenbio, dilution 1:1500), anti‐LIAS (11577‐1‐AP, Proteintech, dilution 1:1000), anti‐FDX1 (12592‐1‐AP, Proteintech, dilution 1:1000), anti‐DLAT (R27216, Zenbio, dilution 1:2000), anti‐HSD11B1 (10928‐1AP, Proteintech, dilution 1:2000), anti‐APOC1 (R389018, Zenbio, dilution 1:1000) and anti‐ATP7B (R389236, dilution 1:1000). The PVDF membrane was washed thrice with TBST and incubated with secondary antibody for 1 h at room temperature. The secondary antibodies included goat anti‐rabbit IgG (ab205718, Abcam, dilution 1:10000) and goat anti‐mouse IgG (ab6789, Abcam, dilution 1:10000). Afterward, the PVDF membrane was washed thrice, and proteins were detected by enhanced chemiluminescence (Amersham Corporation, Arlington Heights, IL, USA). The intensity of each band was quantified by Image J. The value of each band density in experimental and control groups was normalized to that of its corresponding GAPDH band (loading control, ratio to GAPDH%).

### ChIP (Chromatin Immunoprecipitation) Assay

ChIP assays were performed using EZ‐Magna ChIP Chromatin Immunoprecipitation Kit (Millipore), as described previously.^[^ ^47^, ^48^ ^]^ Briefly, trophoblast cells (2 × 10^7^ cells) were digested using trypsin, cross‐linked in PBS containing 1% formaldehyde at room temperature for 15 min, and then quenched with 125 mM glycine for 5 min. DNAs were extracted and sonicated to generate DNA fragments with 300–600 bp, as validated by 2.5% agarose gel electrophoresis. Subsequently, the resulting mixture was incubated with FOXO1 antibody (18592‐1‐AP, Proteintech, dilution 1:2000) overnight at 4 °C, with an equal weight of IgG antibody as a negative control. Protein A/G magnetic beads were then added and incubated at 4 °C for another 4 h to form the bead‐protein‐DNA complex. After elution and de‐crosslinking, the precipitated DNA fragments were extracted using DNA extraction reagent (phenol/chloroform/isoamyl alcohol). The promoter regions of STEAP4 were amplified by RT‐qPCR with specific primers (sequences in Table S4, Supporting Information).

### Luciferase Reporter Assay

Luciferase reporter assays were performed as described previously.^[^ ^72^, ^92^ ^]^ Briefly, wild‐type (wt, 5′‐ ATATGTTACCAGGACAAGACCTCTGGGGAG‐3′) or mutant (mut, 5′‐ CTCCCCAGAGGTCTTGTCCTGGTAACATAT ‐3′) sequence in the STEAP4 promoter region was fused into luciferase pGL3‐basic reporter vector (Promega, Madison, USA) to construct pmirGLO‐STEAP4‐wt/‐mut (sequences in Table S4, Supporting Information). Human trophoblast Swan 71 cells were seeded into 24‐well plates and were co‐transfected with 100 ng pmirGLO‐STEAP4‐wt/‐mut and exposed to 10 mM LiCl for 48 h according to the manufacturer's instructions. Cells were lysed using passive lysis buffer (Promega Corporation), and the firefly luciferase activity in each well was measured using Dual‐Luciferase Reporter Assay System (Promega) according to the manufacturer's protocols.

### Structural and Energy Analysis of Li‐Exposed FOXO1 and Li‐Exposed FOXO1 Bound to STEAP4 Promoter Region—Selection of FOXO1 Region and STEAP4 Promoter Region

Human protein structure FOXO1_HUMAN (UniProt ID: Q12778) was selected from the UniProt database. The DNA‐binding domain of FOXO1 contained amino acid residues from 151 to 249. *STEAP4* promoter region 5′‐TGAAAGACATGGGGTCTGACCAACC‐3′.

### Structural and Energy Analysis of Li‐Exposed FOXO1 and Li‐Exposed FOXO1 Bound to STEAP4 Promoter Region—Molecular Dynamics (MD) Simulations

The structure of the FOXO1 protein was constructed by Alphafold3. The 3D structure was visualized by PyMOL v2.5.4, and its 2D structures were analyzed using PDBsum. The structures of FOXO1 protein or FOXO1 bound to the STEAP4 promoter region in the presence of 10 mM LiCl (with 10 mM NaCl as a control group) were analyzed by MD simulations (within 100 ns) using GROMACS 2020.6. Protein parameters and topology files were generated using Amber03 force field. In the simulation box, periodic boundary conditions were applied, and FOXO1 protein or FOXO1‐DNA complexes were centered in a cubic box (minimum 1.0 nm edge distance) with TIP3P water as solvent. Charge neutrality was maintained by replacing the solvent with 10 mM NaCl or LiCl. Energy minimization was conducted via the steepest descent algorithm to eliminate steric clashes and optimize solvent orientation.

The molecular dynamics pre‐equilibration was conducted in two consecutive phases. First, the NVT ensemble (constant particle number, volume, and temperature) at 300 K for 100 ps obtained the stabilized temperature. Second, the NPT ensemble (constant particle number, pressure, and temperature) at 1 bar for 100 ps obtained the stabilized pressure.

Production MD simulations were executed under isothermal‐isobaric conditions (300 K, 1 bar) within 100 ns using the leapfrog algorithm. For post‐simulation, trajectories were aligned to the protein backbone, and root mean square deviation (RMSD) was analyzed.

### Structural and Energy Analysis of Li‐Exposed FOXO1 and Li‐Exposed FOXO1 Bound to STEAP4 Promoter Region—Ramachandran Plot Assessment

The Ramachandran plot (generated using the PyMod module in PyMOL v2.5.4) evaluated the quality of the 3D structural model of the FOXO1 protein. This model exhibited strong overall quality with 48.4% residues in the most favored regions (red), 27.9% in additional allowed regions (yellow), 9.1% in generously allowed regions (light yellow), and 14.5% in disallowed regions (white).

### Structural and Energy Analysis of Li‐Exposed FOXO1 and Li‐Exposed FOXO1 Bound to STEAP4 Promoter Region—FOXO1 Protein 2D Structure Annotation

The secondary structures of FOXO1 or LiCl (or NaCl)‐exposed FOXO1 were analyzed using PDBsum. Sequences were represented by single‐letter amino acid codes. Secondary structural elements included α‐helices (labeled H1, H2, etc., depicted as purple cylinders), β‐sheets (strands labeled A, B, etc., shown as purple arrows), and conformational motifs (β‐turns, γ‐turns, and β‐hairpins).

### Structural and Energy Analysis of Li‐Exposed FOXO1 and Li‐Exposed FOXO1 Bound to STEAP4 Promoter Region—3D Structural Analysis and Visualization

AlphaFold3 was employed to predict the 3D structures of FOXO1 or the FOXO1‐DNA complex. PyMOL v2.5.4 was used for detailed 3D visualization and binding interface analysis. The 3D structure of FOXO1 protein contained α‐helices in red, β‐sheets in yellow, and random coils in green. The 3D structure of FOXO1‐DNA complex contained stable interactions, such as H‐bonds (formed between polar donors (e.g., N‐H) and acceptors (e.g., O, N), energy range in 10–40 kJ mol^−1^), salt bridges (formed between oppositely charged residues, distance ≤ 5.5 Å, energy range in 3–13 kJ mol^−1^), hydrophobic interactions (aggregation of hydrophobic groups), π‐π Stacking (weak interactions of aromatic rings between electron‐rich and electron‐deficient systems), π‐cation interactions (interactions between π‐systems (e.g., aromatic rings) and cations (e.g., positively charged residues)).

*Binding Energy Calculation*: The binding energies (kcal/mol) of FOXO1‐STEAP4 promoter region were analyzed by gmx_MMPBSA v1.6.1 (single trajectory method).

### Villous Tissues and Serum Samples

We newly recruited 50 patients with unexplained miscarriage (UM group) and 50 women with elective miscarriage to terminate their unwanted pregnancies, which were considered as healthy control (HC) group, in the age between 25 and 30 and with 6–10 weeks of gestation, as the similar methods described previously^[^ ^42^, ^43^, ^49^ ^]^ Any women with clinically known causes of miscarriage were excluded, such as cervical incompetence, chromosome abnormalities, endocrine or metabolic diseases, virus or bacterial infections, as described previously.^[^ ^47^, ^48^, ^72^ ^]^ HC group had previous successful pregnancies. HC and UM women did not receive any treatment. The criteria of pregnancy were considered by the professional doctors according to factors such as missed menstruation, morning sickness, β‐hCG level, and early pregnancy factors. Miscarriage is defined by the World Health Organization (WHO) as the loss of an embryo or fetus weight < 500 g or before 20 weeks of gestation, including spontaneous loss or medically artificial miscarriage.^[^ ^93^ ^]^ The characteristic parameters of these HC and UM women were listed in Table S5 (Supporting Information), including baseline characteristic (age, body mass index, education, incoming, residence), clinical information (gravidity, gestational days, RBC, WBC, Hb), and lifestyle (smoking, drinking) in the period of three month before miscarriage operation. All this information was obtained from medical records. Villous tissue and peripheral blood samples were collected on the same day of the miscarriage operation at the hospital. Villous tissue samples were separated from the decidua tissues in the mid‐connection part of the placenta. After washing with sterile saline, villous tissue samples were stored at −80 °C before RNA or protein extraction. For RNA or protein extraction, ≈30 mg villous tissues were homogenized in 600 µL Trizol reagent (Invitrogen) for RNA extraction or in 600 µL RIPA lysis buffer (Thermo Fisher Scientific) containing protease inhibitor cocktail for protein extraction using Silica beads (107735, Merck, Darmstadt, Germany) via shaking for a 1‐min burst using a TissueLyser LT instrument (Qiagen). Peripheral blood samples were collected in BD Vacutainer SST tubes. Serum samples were isolated within 30 min by centrifugation and were stored in aliquots at −80 °C until further use. The contents of multiple metal elements in serum were determined by ICP‐MS. The experiment protocols were approved by the Ethics Committee of the Eighth Affiliated Hospital of Sun Yat‐sen University. Written informed consents were collected from all the participants before enrollment.

### Determination of Li and Cu Element Content in Serum or Villous Tissue Samples by Inductively Coupled Plasma‐Mass Spectrometry (ICP‐MS)

Serum samples or villous tissue samples were evaporated, dissociated, atomized, and ionized in a plasma axial channel at high temperatures (≈8000 K). The charged positive ions entered the mass spectrometer through the ion acquisition system, and the elements were separated according to their mass‐to‐charge ratios. Within a certain concentration range, the intensity of the element was proportional to its concentration. Li levels were determined using an ICP mass spectrometer (PerkinElmer Inc., model NexION 350X, Massachusetts, USA) based on a standard curve.^[^ ^94^ ^]^ Li levels were expressed as µg/L in serum samples and µg/kg in villous tissues. Cu element in serum was also determined by ICP‐MS. Cu levels were expressed as µg/L in serum samples.

### Determination of Cu+ ion Levels in Human Trophoblast Swan 71 Cells, Villous Tissues, and Mouse Placental Tissues

Trophoblast Swan 71 cells were treated with 0, 0.02, 5, 10, or 20 mM LiCl, with 0 or 10 mM LiCl and 50 nM siRNA (si‐FOXO1 or si‐STEAP4), or with 1 µg pcDNA3.1‐STEAP or pcDNA3.1‐FOXO1. Free Cu^+^ levels in Swan 71 cells, villous tissues, or mouse placental tissues were detected using Coppersensor‐1 (CS1) probe (T40996, Targetmol).^[^ ^95^ ^]^ Cells (2 × 10^4^ cells) were incubated with 5 µM Coppersensor‐1 probe at 37 °C for 15 min. Then, fluorescence was measured at 543 nm on a fluorescence microscope (Leica, Germany). Women's villous tissues or mouse placental tissues were made into cryosections. Then, these sections were incubated with 5 µM Coppersensor‐1 probe in PBS at 37 °C for 15 min for the determination of fluorescence at 543 nm. Fluorescence signals in six digital images per well or section were recorded and analyzed using ImageJ. The unexposed Swan 71 cells were used as control cells. The levels of free Cu^+^ ions were expressed as the mean fluorescence intensity and were normalized against the levels in the control group. Tissue sections without Coppersensor‐1 treatment were considered as baseline assays to exclude the potential autofluorescence. All experiments were replicated thrice.

### Li‐Exposed Mouse Models

Li‐exposed mouse models were constructed as a similar method described previously.^[^ ^47^, ^48^, ^72^ ^]^ Briefly, female C57BL/6 mice (aged 6–8 weeks, Charles River Company, Beijing, China) were kept in standard environmental conditions (featuring a 12 h light/dark cycle and a temperature of 22 °C) and mated with male mice overnight. The appearance of a vaginal copulation plug was considered as day 1 (D1) of pregnancy, which was further monitored by weighing the mouse body every day.^[^ ^90^ ^]^ The pregnant mice were randomly divided into five groups (*n* = 6 per group) and were orally given varying doses of LiCl in saline solution every day from D1 to D13 (Model I). A study showed that Li intake dose in the residents under a normal environment in the Canary Islands, Spain, was ≈3.674 mg^−1^ d^−1[^ ^96^ ^]^ (i.e., 0.05 mg^−1^ kg^−1^ d^−1^ Li based on an average adult weight of 70 kg). According to the body surface area coefficient (mouse/human = 12), the real intake dose corresponded to 0.6 mg^−1^ kg^−1^ d^−1^ Li in the mouse (i.e., 3.6 mg^−1^ kg^−1^ d^−1^ LiCl), which could be considered as a real environment exposure dose (REED). Clinically, the doses of Li_2_CO_3_ used for treatment against bipolar disorder patients were in the range from ≈700 to 1800 mg^−1^ d^−1^,^[^ ^97^ ^]^ corresponding to 100–200 mg^−1^ kg^−1^ d^−1^ LiCl in mice. Considering the doses used in literature, short lifespan of mice, and the feasibility of mouse model assays, in this study, 0, 3.6, 20, 100, or 200 mg^−1^ kg^−1^ d^−1^ LiCl were chosen in this mouse model, corresponding to control, 1‐fold, 5.6‐fold (median dose), 28‐fold (therapeutic dose), or 56‐fold (adverse outcome exposure dose) REED of Li. The same volume of saline solution was used in the control group.

We also constructed a miscarriage intervention model using TTM (a typical inhibitor of cuproptosis, Model II) and two miscarriage treatment models with AS1842856 (a suppressor of FOXO1, Model III) or Forskolin (a suppressor of STEAP4, Model IV), as a similar method described previously.^[^ ^15^, ^61^, ^98^ ^]^ In Model II, pregnant mice were randomly divided into four groups (*n* = 6 per group) and treated with 1) saline solution, 2) 200 mg^−1^ kg^−1^ d^−1^ LiCl, 3) 200 mg^−1^ kg^−1^ d^−1^ LiCl and 2% DMSO, or 4) 200 mg/kg/d LiCl and 10 mg^−1^ kg^−1^ 3d^−1^ TTM in DMSO. In Model III, pregnant mice were randomly divided into four groups (*n* = 6 per group) and treated with 1) saline solution, 2) 200 mg^−1^ kg^−1^ d^−1^ LiCl, 3) 200 mg^−1^ kg^−1^ d^−1^ LiCl and 2% DMSO, or 4) 200 mg^−1^ kg^−1^ d^−1^ LiCl and 3.5 mg^−1^ kg^−1^ 3d^−1^ AS1842856 in DMSO. In Model VI, pregnant mice were randomly divided into four groups (*n* = 6 per group) and treated with 1) saline solution, 2) 200 mg^−1^ kg^−1^ d^−1^ LiCl, 3) 200 mg^−1^ kg^−1^ d^−1^ LiCl and 2% DMSO, or 4) 200 mg^−1^ kg^−1^ d^−1^ LiCl and 2 mg^−1^ kg^−1^ 3d^−1^ Forskolin in DMSO. Mice were daily given LiCl by oral gavage from D1 to D13. TTM has been widely used as an inhibitor of cuproptosis in various cell and animal studies.^[^ ^99^ ^]^ AS1842856 was a suppressor of FOXO1^[^ ^79^, ^80^ ^]^ and Forskolin was a suppressor of STEAP4,^[^ ^83^ ^]^ which have been widely used in cell and animal studies. TTM, AS1842856, or Forskolin in DMSO was intraperitoneally injected into mice once per three days from D1 to D13.

On D14, all mice in four mouse models were euthanized by injection with Nembutal (100 mg^−1^ kg^−1^) for the collection of the uterus. The embryo resorption was identified by a smaller or darker appearance than the viable and pink healthy embryos.^[^ ^100^ ^]^ The miscarriage rate in each mouse and the average miscarriage rate in each group were calculated by the number of adsorbed embryos / (the number of normal embryos + the number of adsorbed embryos). RNAs and proteins were extracted from a random placenta in each of the mouse uteri for RT‐qPCR and Western blot analysis, respectively. The gene conservation and homology analysis were conducted using the UCSC Blast‐like alignment tool (BLAT) genome browser (https://genome.ucsc.edu/cgi‐bin/hgBlat.^[^ ^101^, ^102^ ^]^ The protein conservation and homology analysis were conducted using Uniprot (https://www.uniprot.org/).^[^ ^103^ ^]^ The animal project has been authorized by the Ethics Committee of the Eighth Affiliated Hospital of Sun Yat‐sen University.

### Statistical Analysis

All experiments were replicated thrice independently with similar results. The measured or calculated data (including the control group) were presented as mean ± SD (standard deviation, *n* = 3). SPSS 26.0 software was used to analyze the data, and GraphPad Prism 9.0 software was used for figure generation. In cellular assays, the number *n* = 3 indicated that all experiments were replicated thrice independently. Data in other groups were normalized against that in the control group. In the animal model, the number *n* = 6 indicated 6 mice in each group. In villous tissue assays, *n* = 50 meant 50 pair of HC and UM villous tissue or serum samples. Levene's test was used to evaluate the homogeneity of variance of the data. Non‐normal data were analyzed by a non‐parametric test (Mann‐Whitney U). Student's t‐test was used to compare differences between two groups. ANOVA and Fisher's least significant difference (LSD) test were used to compare differences among multiple groups. The qualitative data were compared using the Chi‐square test. The correlation analysis of the relative expression levels was performed using Pearson correlation analysis. The odds ratio (OR) of each variable and its 95% confidence interval (95% CI) were analyzed by univariate logistic regression. The OR and 95% CI by adjusting for potential confounders were analyzed by multivariate logistic regression. Receiver Operating Characteristic was plotted by GraphPad Prism 9.0 software. When *p* < 0.05, the difference was considered to be statistically significant.

## Conflict of Interest

The authors declare no conflict of interest.

## Author Contributions

S.X., Y.L., and Y.S. contributed equally to this work co‐first authors. H.Z., S.X., Y. L., and Y.S. conceived the project, designed the experiments, analyzed results, and wrote the manuscript. S.X. and Y. L. performed the majority of experiments. X.Y. did molecular stimulation. Y.S. and Q.F. participated in mouse experiments. Q.K., Y.W., and H.Y. made minor experiments. J.N., Z.Z., Y.L., J.Y., C.T., and Y.C. contributed constructive comments and data analysis.

## Supporting information



Supporting Information

## Data Availability

All data and materials presented in this manuscript are available from the corresponding author (H. Zhang) upon a reasonable request under a completed Material Transfer Agreement. Any additional information required to reanalyze the data reported in this work paper is available from the corresponding author upon request.
