# Supplementary material for: Lithium Exposure Causes Trophoblast Cuproptosis by Upregulating FOXO1/STEAP4 Axis in Unexplained Miscarriage
Source: Adv Sci (Weinh). 2025 Aug 25;12(38):e02139. doi: 10.1002/advs.202502139 (PMC12520556; doi:10.1002/advs.202502139)
Supplement: Supplementary file 1 — Supporting Information [file ADVS-12-e02139-s001.docx]

Supporting information

**Table S1. DNA sequence used for construction of pcDNA3.1 overexpression plasmids.**

| **Plasmid name** | **Gene name** | **Sequence region** |
| --- | --- | --- |
| pcDNA3.1-STEAP4 | *STEAP4* | CDS region (NM_024636.4) |
| pcDNA3.1-FOXO1 | *FOXO1* | CDS region (NM_002015.4) |

**Table S2. RNA sequences used for RNA transfection.**

| Name | Sense (5'-3') | Antisense (5'-3') |
| --- | --- | --- |
| si1-STEAP4 | AAAGCAAAGAGUGAUGGAUA(dT)(dT) | UAUCCAUCACUCUUUGCUU(dT)(dT) |
| si2-STEAP4 | AUGGCUAUUUCCAUUCCAA(dT)(dT) | UUGGAAUGGAAAUAGCCAU(dT)(dT) |
| si3-STEAP4 | AUGGAGAAAACUUGUAUAGAU(dT)(dT) | AUCUAUACAAGUUUUCUCCAU(dT)(dT) |
| si1-FOXO1 | CUGCAUCCAUGGACAACAA(dT)(dT) | UUGUUGUCCAUGGAUGCAG(dT)(dT) |
| si2-FOXO1 | CCAGAUGCCUAUACAAACA(dT)(dT) | UGUUUGUAUAGGCAUCUGG(dT)(dT) |
| si3-FOXO1 | AUUAUGACGAAUUGAAUUC(dT)(dT) | GAAUUCAAUUCGUCAUAAU(dT)(dT) |
| si1-NC | UUCUCCGAACGUGUCACGUTT | ACGUGACACGUUCGGAGAATT |

**Table S3. Primer sequences used for RT-qPCR analysis.**

| **Species** | **Gene** | **Forward (5'-3')** | **Reverse (5'-3')** |
| --- | --- | --- | --- |
| Human | APOC1 | TCCAGTGCCTTGGATAAGCTG | GGCTGATGAGTTCCCGAGC |
|  | HSD11B1 | CTCAGTTACGTGGTCCTGACT | GAGGAGACGACAACAATGCTT |
|  | STEAP4 | GGCTTTGGGAATACTTGGGTT | TGGACAAATCGGAACTCTCTCC |
|  | FOXO1 | GGATGTGCATTCTATGGTGTACC | TTTCGGGATTGCTTATCTCAGAC |
|  | DLAT | CGGAACTCCACGAGTGACC | CCCCGCCATACCCTGTAGT |
|  | FDX1 | TTCAACCTGTCACCTCATCTTTG | TGCCAGATCGAGCATGTCATT |
|  | LIAS | CAGCCCAGTCAGACCGTTAAG | TTTCTGGCGTTTTAGGTTTCCT |
|  | SLC31A1 | GGGGATGAGCTATATGGACTCC | TCACCAAACCGGAAAACAGTAG |
|  | ATP7B | GGGGACGATGCCTGAACAG | GCCGGGCAAAGCAAGTTTAG |
| Mouse | Steap4 | GGGAAGTCACTGGGATTGAAAA | CCGAATAGCTCAGGACCTCTG |
|  | Foxo1 | CCCAGGCCGGAGTTTAACC | GTTGCTCATAAAGTCGGTGCT |

**Table S4. Primer sequences used in** **various RT-qPCR assays.**

| **Species** | **Gene** | **Forward(5'-3')** | **Reverse(5'-3')** |
| --- | --- | --- | --- |
| Chip primers | |  |  |
| Human | STEAP4 | TGAAAGACATGGGGTCTGACCAACC | GAGGCCTGTTCCTCCCATGGAAA |
| Mouse | Steap4 | GCTCTTTCCTCCACGTTGTCAT | CAACAATACCTGCTTACACATGCAG |
| Cloning primers | |  |  |
| Human | STEAP4 | ATATGTTACCAGGACAAGACCTCTGGGGAG | ATATAAGCTTGGTAGCCAGCCTGTGCTCTG |

**Table S5. Univariate analysis of various variables in HC and UM villous tissues^a^**

| **Variable** | **HC***^b^* **(n=50)** | **UM***^c^* **(n=50)** | ***P****^d^* |
| --- | --- | --- | --- |
| Li levels in serum (μg/L) | 6.130 (4.680, 7.400) | 12.26 (6.470, 15.18) | < 0.001 |
| Li levels in villous tissue (μg/kg) | 9.740 ± 5.580 | 14.60 ± 5.206 | < 0.001 |
| Age | 28.60 ± 1.578 | 28.00 ± 1.884 | 0.087 |
| Gestational days (d) | 47.41 ±1.638 | 47.00 ± 2.104 | 0.277 |
| BMI*^e^* | 23.85 ± 0.694 | 23.54 ± 0.900 | 0.054 |
| RBC*^f^* | 5.589 ± 0.040 | 5.594 ± 0.148 | 0.802 |
| WBC*^g^* | 6.895 ± 0.485 | 7.020 ± 0.544 | 0.226 |
| Hb*^h^* | 156.2 ± 1.082 | 156.2 ± 2.164 | 0.963 |
| **Education**  < High school  ≥ High school | 16  34 | 18  32 | 0.673 |
| **Smoking in the past 3 months**  seldom  1-4 times per week  ≥ 4 times per week | 35  6  9 | 33  6  11 | 0.879 |
| **Drinking in the past 3 months**  seldom  1-4 times per week  ≥ 4 times per week | 29  10  11 | 36  6  8 | 0.328 |
| **Residence**  rural  urban | 18  32 | 15  35 | 0.523 |
| Copper levels in serum | 1431 ± 418.3 | 1269 ± 392.4 | 0.121 |
| Copper levels in villous tissue | 1015 ± 453.8 | 886.7 ± 345.9 | 0.113 |

Abbreviation: OR, Odds ratio; CI, Confidence interval

*^a^*mean ± standard deviation or median ± IQR (n =50 in each UM or HC group).

*^b^*HC: healthy control group.

*^c^*UM: unexplained miscarriage group.

*^d^*Student's t-test.

*^e^*BMI: Body Mass Index.

*^f^*RBC: Red blood cell.

*^g^*WBC: White blood cell.

*^h^*Hb: Hemoglobin.

**Table S6. Multivariate logistic regression analysis of Li levels in serum by adjusting age, BMI, education, household income, smoking, drinking, and residence.**

| **Variable** | **Adjusted OR** | **95% CI** | **P-value*^d^*** |
| --- | --- | --- | --- |
| Age | 0.538 | 0.335, 0.866 | 0.011 |
| BMI | 0.563 | 0.264, 1.202 | 0.138 |
| Education | 0.550 | 0.144, 2.109 | 0.383 |
| Household income | 4.863 | 1.15, 20.605 | 0.032 |
| **Smoking** |  |  |  |
| seldom | 1.000 |  | 0.164 |
| 1-4 times per week | 0.076 | 0.005, 1.169 | 0.065 |
| ≥ 4 times per week | 0.453 | 0.087, 2.363 | 0.348 |
| **Drinking** |  |  |  |
| seldom | 1.000 |  | 0.263 |
| 1-4 times per week | 0.384 | 0.083, 1.769 | 0.219 |
| ≥ 4 times per week | 0.311 | 0.057, 1.680 | 0.175 |
| Residence | 6.021 | 1.377, 26.327 | 0.017 |
| Li levels in serum | 1.860 | 1.441, 2.400 | < 0.0001 |

Adjusting for age, BMI, education, household income, smoking, drinking, and residence.

**Table S7. Multivariate logistic regression analysis of Li levels in villous tissues by adjusting age, BMI, education, household income, smoking, drinking, and residence.**

| **Variable** | **Adjusted OR** | **95% CI** | **P-value** |
| --- | --- | --- | --- |
| Age | 0.686 | 0.485, 0.970 | 0.033 |
| BMI | 0.456 | 0.239, 0.870 | 0.017 |
| Education | 1.064 | 0.355, 3.195 | 0.911 |
| Household income | 2.620 | 0.854, 8.040 | 0.092 |
| **Smoking** |  |  |  |
| seldom | 1.000 |  | 0.322 |
| 1-4 times per week | 1.290 | 0.294, 5.652 | 0.736 |
| ≥ 4 times per week | 2.622 | 0.747, 9.208 | 0.132 |
| **Drinking** |  |  |  |
| seldom | 1.000 |  | 0.680 |
| 1-4 times per week | 0.636 | 0.166, 2.431 | 0.508 |
| ≥ 4 times per week | 0.620 | 0.165, 2.332 | 0.480 |
| Residence | 2.121 | 0.713, 6.306 | 0.176 |
| Li levels in villous tissue | 1.234 | 1.113, 1.368 | < 0.0001 |

Adjusting for age, BMI, education, household income, smoking, drinking, and residence.

**Table S8. Sequence conservation among various species.**

|  |  |  | Human | Rhesus | Mouse | Dog | Elephant |
| --- | --- | --- | --- | --- | --- | --- | --- |
| mRNA | SLC31A1 | Per.Ident^a^ | 100% | 96.0% | 88.9% | 88.7% | 88.7% |
|  | LIAS | Per.Ident^a^ | 100% | 95.4% | 89.9% | 93.9% | 91.3% |
|  | FDX1 | Per.Ident^a^ | 100% | 95.2% | 85.3% | 90.7% | 88.3% |
|  | DLAT | Per.Ident^a^ | 100% | 96.9% | 89.9% | 90.5% | 88.2% |
|  | FOXO1 | Per.Ident^a^ | 100% | 94.9% | 90.1% | 90.4% | 89.0% |
|  | STEAP4 | Per.Ident^a^ | 100% | 94.8% | 84.4% | 86.7% | 86.7% |
| Protein | SLC31A1 | Per.Ident^a^ | 100% | 99.0% | 96.3% | 94.0% | 99.4% |
|  | LIAS | Per.Ident^a^ | 100% | 99.0% | 93.7% | 96.0% | 96.0% |
|  | FDX1 | Per.Ident^a^ | 100% | 96.8% | 90.3% | 89.6% | 91.2% |
|  | DLAT | Per.Ident^a^ | 100% | 98.2% | 90.1% | 89.8% | 90.4% |
|  | FOXO1 | Per.Ident^a^ | 100% | 94.7% | 94.7% | 94.9% | 95.2% |
|  | STEAP4 | Per.Ident^a^ | 100% | 97.2% | 83.3% | 88.2% | 91.4% |

^a^Percent Identity reflects the degree of consistency between human sequence and the sequences in other species. Higher value means better conservation.

**Table S9.**

| Key residue | Total Energy (kcal/mol) | | Δ Energy (kcal/mol) |
| --- | --- | --- | --- |
|  | Complex of FOXO1-MBOAT1-NaCl | Complex of  FOXO1-MBOAT1-LiCl |  |
| Total | -129.77 | -167.19 | -37.42 |
| LYS-151 | -3.72 | -7.47 | -3.75 |
| ARG-156 | -0.36 | -1.63 | -1.27 |
| ARG-157 | -3.21 | -11.34 | -8.13 |
| ASN-158 | -0.85 | -2.4 | -1.55 |
| TYR-165 | -1.91 | -3.39 | -1.48 |
| LEU-183 | -0.45 | -2.27 | -1.82 |
| ASN-204 | -0.37 | -3.13 | -2.76 |
| SER-212 | -0.14 | -1.81 | -1.67 |
| ARG-214 | -5.84 | -7.04 | -1.2 |
| SER-218 | -0.91 | -2.14 | -1.23 |
| ARG-225 | -3.22 | -4.62 | -1.4 |
| TRP-237 | -0.32 | -1.81 | -1.49 |

Fig. S1.

(A) A directed acyclic graph represented the effects of potential confounders (pink circles) on the association between primary cause (Li exposure) and outcome (unexplained miscarriage), as analyzed by DAGitty software. Green lines represented causal path and red lines represented biasing paths. (B) ICP-MS analysis of the element levels of Zn, Ni, Mn, Fe, Co, As, Cd, Pb, or Cu in HC and UM serum samples (each n=18).

Fig. S2.

(A) Volcanic chart of mRNA sequencing data (including ATP7B mRNA levels) of 10 vs 0 mM LiCl-exposed Swan 71 cells, UM vs HC villous tissues, and 28 vs 0-fold REED of LiCl-exposed mouse placenta. (B) RT-qPCR analysis of ATP7B mRNA levels in LiCl-exposed Swan 71 cells (n = 3). (C-D) The protein levels of ATP7B in LiCl-exposed Swan 71 cells and its relative quantification (n = 3).

Fig. S3.

(A-B) Forest plots of odds ratios (ORs) of LIAS, SLC31A1, FDX1, FOXO1, and STEAP4 protein by adjusting for all these confounders and their 95% confidence interval (95% CI) in HC and UM groups. (C-E) Pearson correlation analysis of the correlation between Li levels in serum and the protein levels of LIAS, SLC31A1, or FDX1 in villous tissues in HC and UM groups (each n = 12). (F) The relative levels of Cu^+^ ions in 0, 5.6, 28, or 56-fold REED of LiCl-exposed mouse placenta, with CS1 fluorescent probe.

Fig. S4.

(A) Volcanic chart of the differentially expressed genes (DEGs) in mRNA sequencing data of 10 vs 0 mM LiCl-exposed Swan 71 cells, UM vs HC villous tissues, and 28 vs 0-fold REED of LiCl-exposed mouse placenta. (B-C) RT-qPCR analysis of APOC1 and HSD11B1 mRNA levels in LiCl-exposed Swan 71 cells (n = 3). (D-E) Western blot analysis of APOC1 and HSD11B1 protein levels in LiCl-exposed Swan 71 cells and their relative quantification. (F) STEAP4 mRNA levels in Swan 71 cells with knockdown or overexpression of STEAP4 (n = 3). (G) The protein levels of STEAP4 in Swan 71 cells with knockdown of STEAP4 and their relative quantification (n = 3). (H) CCK8 assay analysis of Swan71 cell viability with overexpression of STEAP4 and with co-treatment TTM, ES, Fer-1, Nec-1, Z-VAD-FMK, 3-MA, or TCEP at 48 h. (I) CCK8 assay analysis of cell viability of 10 mM LiCl-exposed Swan 71 cells treated with TTM, ES, Fer-1, Nec-1, Z-VAD-FMK, 3-MA, or TCEP at 48 h.

Fig. S5.

(A) The mRNA stability assay analysis of STEAP4 mRNA levels in 10 mM LiCl-exposed Swan 71 cells with actinomycin D (5 μg/mL) treatment for 0-8 h. (B) FOXO1 mRNA levels in LiCl-exposed Swan 71 cells (n = 3). (C-D) FOXO1 mRNA and protein levels in Swan 71 cells with knockdown or overexpression of FOXO1 and its relative quantification (n = 3). (E-F) STEAP4 mRNA and protein levels in Swan 71 cells with knockdown or overexpression of FOXO1 and its relative quantification (n = 3). (G-H) FOXO1 and STEAP4 mRNA levels in LiCl-exposed Swan 71 cells with FOXO1 knockdown (n = 3). (I-J) The 3D protein structure of FOXO1 was obtained by MD simulations using GROMACS within 100 ns, as visualized by PyMOL v2.5.4. The 2D structures of FOXO1 were analyzed using PDBsum. FOXO1 protein contained 64 amino acid residues in α-helices in red, 15 residues in β-sheets in yellow, 576 residues in random coils in green. (K) The Ramachandran plot (generated using PyMod module in PyMOL v2.5.4) analysis of the quality of of FOXO1 protein 3D structure. This model exhibited strong overall quality with 48.4% residues in the most favored regions (red), 27.9% in additional allowed regions (yellow), 9.1% in generously allowed regions (light yellow), and 14.5% in disallowed regions (white). (L) The 3D structure of the complex between the DNA binding region (residues 151-249) of FOXO1 in green and STEAP4 promoter region (5’-TGAAAGACATGGGGTCTGACCAACC-3’) in blue contained 4 hydrophobic interaction, 14 H-bonds, 1 salt bridges, and 1 π-Stacking interaction. (M) The 3D structure of the complex between the DNA binding region of FOXO1 in green and STEAP4 promoter region in blue in the presence of 10 mM NaCl contained 2 hydrophobic interaction, 23 H-bonds, 7 salt bridges, and 1 π-Stacking interaction. (N) The 3D structure of the complex between the DNA binding region of FOXO1 in green and STEAP4 promoter region in blue in the presence of 10 mM LiCl contained 4 hydrophobic interaction, 25 H-bonds, 6 salt bridges, and 1 π-Cation interaction.

Fig. S6.

(A-B) Schematic diagram of LiCl-exposed mouse model with AS1842856 or Forskolin treatment. Pregnant mice (each n = 6) were treated with saline or 200 mg/kg/d LiCl by oral gavage and with AS1842856 (3.5 mg/kg/3d) by intramuscular injection or with Forskolin (2 mg/kg/3d) by oral gavage for continuous 13 days. (C-D) The protein levels of FOXO1 and STEAP4 in mouse placental chorionic trophoblast cells with AS1842856 or Forskolin treatment and their relative quantification.
